# Comprehensive Evolutionary Analysis of the Major RNA-Induced Silencing Complex Members

**DOI:** 10.1038/s41598-018-32635-4

**Published:** 2018-09-21

**Authors:** Rui Zhang, Ying Jing, Haiyang Zhang, Yahan Niu, Chang Liu, Jin Wang, Ke Zen, Chen-Yu Zhang, Donghai Li

**Affiliations:** State Key Laboratory of Pharmaceutical Biotechnology, Jiangsu Engineering Research Center for MicroRNA Biology and Biotechnology, Nanjing Advanced Institute for Life Sciences (NAILS), School of Life Sciences, Nanjing University, Nanjing, Jiangsu 210023 P.R. China

## Abstract

RNA-induced silencing complex (RISC) plays a critical role in small interfering RNA (siRNA) and microRNAs (miRNA) pathways. Accumulating evidence has demonstrated that the major RISC members (AGO, DICER, TRBP, PACT and GW182) represent expression discrepancies or multiple orthologues/paralogues in different species. To elucidate their evolutionary characteristics, an integrated evolutionary analysis was performed. Here, animal and plant AGOs were divided into three classes (multifunctional AGOs, siRNA-associated AGOs and piRNA-associated AGOs for animal AGOs and multifunctional AGOs, siRNA-associated AGOs and complementary functioning AGOs for plant AGOs). Animal and plant DICERs were grouped into one class (multifunctional DICERs) and two classes (multifunctional DICERs and siRNA-associated DICERs), respectively. Protista/fungi AGOs or DICERs were specifically associated with the siRNA pathway. Additionally, TRBP/PACT/GW182 were identified only in animals, and all of them functioned in the miRNA pathway. Mammalian AGOs, animal DICERs and chordate TRBP/PACT were found to be monophyletic. A large number of gene duplications were identified in AGO and DICER groups. Taken together, we provide a comprehensive evolutionary analysis, describe a phylogenetic tree-based classification of the major RISC members and quantify their gene duplication events. These findings are potentially useful for classifying RISCs, optimizing species-specific RISCs and developing research model organisms.

## Introduction

RNA-induced silencing complex (RISC) plays an important role in small interfering RNA (siRNA)- and microRNA (miRNA)-mediated gene regulation^1–6^. The siRNA pathway regulates target gene expression, provides antiviral responses, and restricts transposons. The miRNA pathway represses target gene expression at the post-transcriptional level and participates in physiological and pathophysiological processes^4–6^. The siRNA/miRNA have found widespread applications as research and clinical techniques, allowing simple yet effective knockdown of target genes of interest and disease therapy^7^. Further, miRNAs are important biomarkers for disease diagnosis and prognosis^8^.

The major members of RISC have been identified and characterized, including *AGO* (*argonaute*), *DICER*, *TRBP* (*TARBP2*), *PACT* (*PRKRA*) and *GW182* (*GAWKY*). The crystal structure of the AGO protein from *Pyrococcus furiosus* at 2.25 Å resolution has been resolved^9^. Dicer, a member of the RNase III family, processes miRNA precursors (pre-miRNAs) to mature miRNAs. Recent studies have demonstrated that AGO/DICER are not required for guide hairpin RNA function^10,11^. Within the RISC loading complex, DICER/TRBP assists in processing pre-miRNAs to mature miRNAs and then load them onto AGO2. AGO2 bound to the mature miRNA constitutes the minimal RISC and may subsequently dissociate from DICER and TRBP. TRBP/PACT found in RISC binds to double-stranded RNAs (dsRNAs) and ensures regular miRNA biogenesis^12,13^. GW182 directly interacts with AGO2, and GW182 proteins are essential for miRNA-guided gene silencing in various organisms. The N-terminal region of GW182 contains multiple GW repeats, which directly associate with an AGO2^14^.

Previously, few phylogenetic analyses of RISC members have been performed. Several conclusions are summarized as follows: (I) vertebrates lack siRNA-class AGO proteins and vertebrate AGOs display low rates of molecular evolution^15^; (II) Dicers might have duplicated and diversified independently and have evolved for various functions in invertebrates^16^; (III) Loquacious identified in insects may be ancestral to both TRBP and PACT; and (IV) significant acceleration in the accumulation of amino acid changes of GW182-binding regions indicates its early origin and adaptive evolution^17^. However, a systematic, integrative evolutionary analysis is still lacking. In our study, we applied improved and integrated bioinformatic softwares/algorithms for investigating the evolution of the major RISC members, and these results provide a novel insight into RISC evolution and RISC-mediated gene regulation.

## Results

### Evolution of RISC-related AGO members

The AGO protein family plays a central role in RISC-mediated gene regulation. AGOs mainly involve four characteristic domains: an N-terminal, PAZ (which is responsible for small RNA binding), Mid and a C-terminal PIWI (which confers catalytic activities) domain (Supplementary Figs S1 and S2)^18,19^. A number of noncoding RNAs are their substrates, including miRNAs, siRNAs and Piwi-interacting RNAs (piRNAs)^20^. Small RNAs guide AGOs to their specific targets through sequence complementarity, which typically leads to silencing of the target mostly by post-transcriptional inhibition or mRNA degradation.

**Figure 1:**
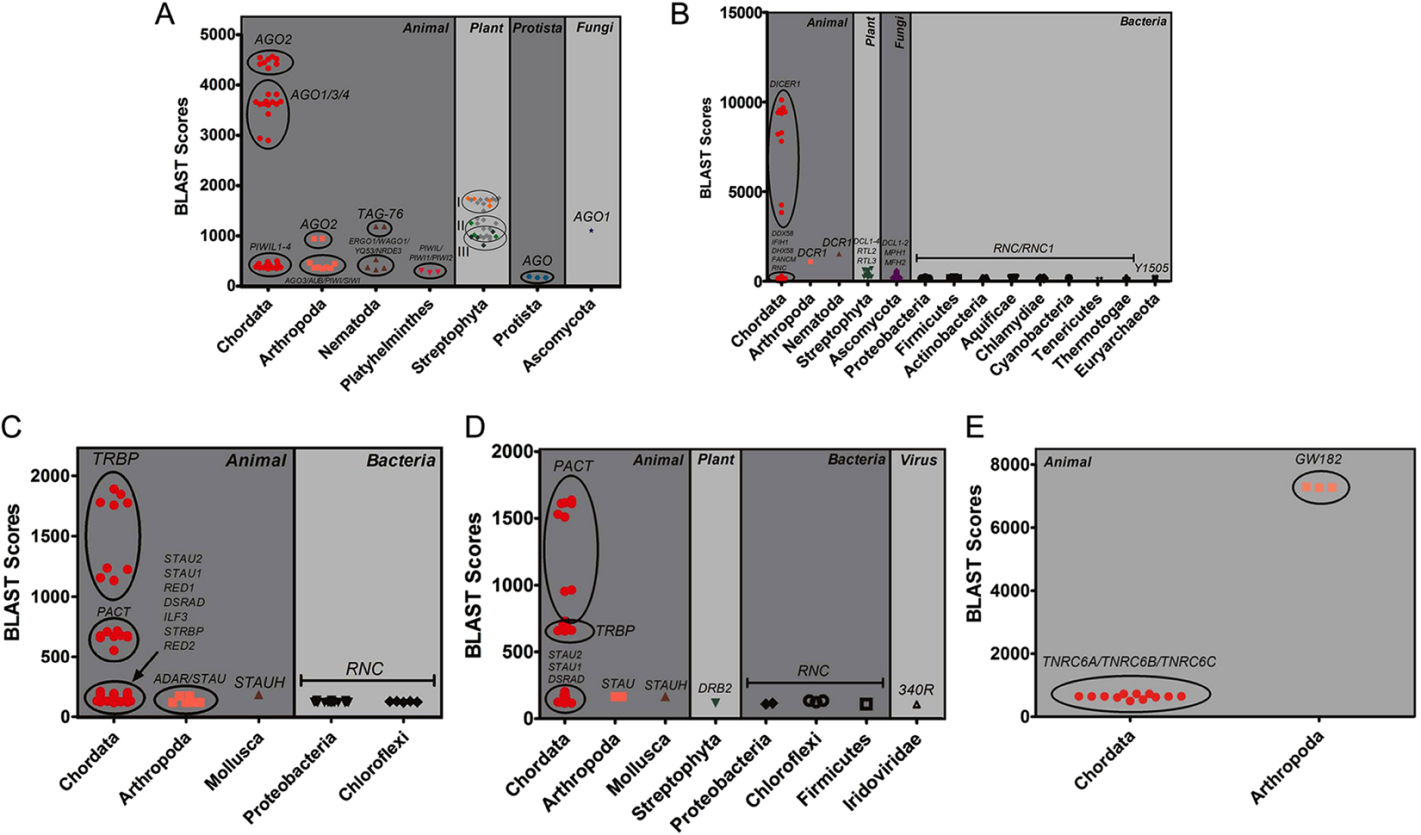
Sequence identities and classifications of the major RISC members in the different species. (**A**) AGOs. *A. thaliana* AGO1/AGO5/AGO10 (orange), AGO2/AGO3/AGO7 (green) and AGO4/AGO6/AGO8/AGO9 (dark green); (**B**) DICERs; (**C**) TRBPs; (**D**) PACTs; (**E**) GW182s.

AGO2 and its homologues have been identified in Chordata, Arthropoda, Nematoda and Platyhelminthes, and a phylogenetic tree of AGOs was constructed (Fig. 1A and Supplementary Fig. S3). According to their RNA-binding characteristics or functions, these AGOs were divided into three classes: (I) multifunctional AGOs; (II) siRNA-associated AGOs; and (III) piRNA-associated AGOs. Class I AGOs contain chordate AGO1–4, in which all human AGOs associate with both siRNAs and miRNAs. As shown in Fig. 1A, chordate *AGO1-4* contain two subclasses: *AGO2* and *AGO1/3/4*. Only AGO2 protein functions as an endonuclease, cleaving mRNA within regions that base pair with perfectly complementary siRNAs or miRNAs. AGO1/3/4 are slicing-incompetent AGOs^21,22^ and remove passenger strands via the bypass mechanism. Class II AGOs specifically bind to siRNAs, which contain Arthropoda (*Drosophila AGO2*) and Nematoda *AGO*s (*TAG-76/ERGO1/WAGO1/YQ53/NRDE3*). *AGO*s in Arthropoda and Nematoda have evolutionary complexities compared with the monophyletic group of mammalian AGOs (Fig. 1A and Supplementary Fig. S3). *Drosophila* AGO2, as a part of the RISC complex, is required for the unwinding of siRNA duplex and subsequent assembly of siRNA into RISC in *Drosophila* embryos. *Drosophila* AGO2 shared a close evolutionary relationship with plant/Chordata AGOs (Supplementary Fig. S3). AGO1 is dispensable for efficient RNAi in *Drosophila* embryos^23^, but it is unreviewed in UniProt and unannotated in Fig. 1A. The phylogenetic tree and Pfam/SMART-based domain structures show that *TAG-76* (*Caenorhabditis elegans, C. elegans*) has the modest similarities with mammalian *AGO*s (Supplementary Fig. S2). Unfortunately, its biological function is still unclear, but other Nematoda proteins participate in the RNAi pathway. *ERGO-1* serves as an *AGO* and functions in the endogenous RNAi pathway^24^. *WAGO1* is a worm-specific *AGO* and silences certain genes, transposons, pseudogenes, and cryptic loci^25^. Uncharacterized *YQ53* with PAZ and PIWI domains shown in Supplementary Fig. S2 has a possible function of endogenous and exogenous RNAi^18^. Another nuclear AGO protein, NRDE3, binds to siRNAs and is required for nuclear RNAi, and thus transports specific classes of small regulatory RNAs to distinct cellular compartments to regulate gene expression^26^. Class III AGOs are composed of chordate *PIWIL1-4*, Arthropoda *AGO3/AUB/ PIWI/SIWI* and Platyhelminthes *PIWIL/PIWI1/PIWI2*. *PIWIL1-4*, the members of the PIWI family, bind to piRNAs and are exclusively expressed in germ-line cells, but other AGOs are ubiquitously expressed in most tissues^27^. In addition, Supplementary Fig. S3 shows that other Arthropoda AGO2-related proteins are close to the subclade of chordate *PIWIL1-4*, and they are directed by piRNAs to cleave transposon transcripts and instruct Piwi to suppress transposon transcription to protect the germline genome in *Drosophila* ovarian germ cells. The members of the PIWI protein family (*PIWIL*, *PIWI1* and *PIWI2*) have also been identified in Platyhelminthes (*Dugesia japonica* and *Schmidtea mediterranea*). They have domains similar to those of animal AGOs and may be required for stem cell function and piRNA biogenesis (Supplementary Figs S2 and S3)^28,29^.

Previous studies have determined three clades of *AGO*s in plants^30^. For example, *Arabidopsis thaliana* (*A. thaliana*) exhibits an equal distribution of its ten members within three clades: (I) *AGO1, AGO5, AGO10*; (II) *AGO2, AGO3, AGO7*; and (III) *AGO4, AGO6, AGO8, AGO9*. Consistent with these results, our analyses showed three major clades, and these plant AGOs were artificially grouped into three classes based on their functions: (I) multifunctional AGOs; (II) siRNA-associated AGOs; and (III) complementary functioning AGOs. However, protein sequence identity-based BLAST scores do not provide the more precise classifications (Fig. 1A and Supplementary Fig. S3). Class I AGOs are *AGO1/AGO5/AGO10* (*A. thaliana*) and *AGO1A/AGO1B/AGO1C/AGO1D/AGO11/AGO12/AGO13/AGO14/AGO17/AGO18/PNH1/MEL1* (*Oryza sativa, O. sativa*). *AGO1* acts in the miRNA and siRNA pathways, and *AGO10* possibly participates in these pathways, at least in some tissues; *AGO5*, localized in both the nucleus and the cytoplasm, binds to small RNAs and regulates RNA-mediated post-transcriptional gene silencing^31^. *PNH1* (*O. sativa*) probably influences the formation of the shoot apical meristem and leaf adaxial cell specification and the RNA silencing pathway^32,33^. *MEL1* probably mediates small RNA-related gene silencing and its function may be performed by another *AGO* in *Arabidopsis*^33^. Class II AGOs contain *AGO2/AGO3/AGO7* (*A. thaliana*) and *AGO2/AGO3/AGO7*(*O. sativa*). *AGO7* acts in the *RDR6/SGS3/DCL4/AGO7* trans-acting siRNA pathway involved in leaf developmental timing. *AGO2/3* lack the DDH motif in the PIWI domain and possibly have similar activities; and *AGO2/AGO3* mutants show no developmental defects. Class III AGOs contain *AGO4/AGO6/AGO8/AGO9* (*A. thaliana*) and *AGO4A/AGO4B/AGO15/AGO16* (*O. sativa*). These AGOs have complementary biological functions. *AGO4* is possibly shared in different nuclear complexes and in a distinct pathway^34^. *AGO6* is required for heterochromatin siRNA and transcriptional gene silencing pathways, and *AGO6* activity is partially redundant with the activity of *AGO4*. *AGO8/9* (*A. thaliana*) mRNAs have a different tissue distribution, and their mutants have no effect on plant phenotypes. Based on sequence identities, there is a clear group of Class I members (orange), but there is an overlapping region between Class II (green) and Class III members (dark green) (Fig. 1A). *AtAGO*s have been studied for decades, but many questions still remain. *OsAGO*s appear to have higher diversities and gene duplications because many isoforms were identified as shown in Fig. 1A and Supplementary Fig. S3.

A single *AGO* containing PIWI domains is identified in the Protista *Giardia intestinalis* (*Giardia lamblia*) genome and regulates variant-specific surface protein (VSP) expression on the surface of each parasite via an RNAi pathway (Supplementary Figs S2 and S3)^35^. Surprisingly, a single *AGO1* with PAZ and Piwi domains from yeast was found in fungi and closely located at the third clade of plants (Supplementary Figs S2 and S3). It may possess sequence specific DNA binding activity, and its detailed role in the RISC could be explored in depth.

### Evolution of RISC-related Dicer members

The RNAi process arises from an interaction between RNA molecules and RISC. Dicer anchors a dsRNA molecule and cuts it to produce short dsRNAs as a primary RNA recognition and processing enzyme in the RNAi process. Dicer is a member of the RNase III family and highly conserved in the evolution. Sequence analyses show that Dicer of each species has similar domains. In most species, the N-terminus of Dicer is an RNA helicase domain followed by a PAZ domain (Supplementary Fig. S1). The C-terminus of Dicer has two RNase III domains and a dsRNA binding domain^36^.

Dicers widely exist in eukaryotes. The current phylogenetic tree of the DICER family shows its independent diversification in animals, plants and fungi (Fig. 1B and Supplementary Fig. S4). Distinct with the phylogenetic tree of animal AGOs, a monophyletic group of animal DICER was found to include Chordata, Arthropoda and Nematoda and one class was functionally generated: multifunctional DICERs. These DICERs possess the dual function of recognizing a hairpin or dsRNA and processing them into mature miRNA-miRNA^*^/siRNA duplexes. It is noteworthy that Arthropoda DICER (*Drosophila DCR1)* is required for miRNA biogenesis, and the unreviewed *Drosophila* DICER paralogue *DCR2* in UniProt may be for generating siRNAs. The helicase motifs of Nematoda DICER (*C. elegans DCR1)* are required for siRNA, but not miRNA, processing^37–40^. Dicer required for piRNA processing remains unidentified. The additional constructed chordate subclade includes *DDX58, DHX58, IFIH1, FANCM* and *RNC* (Fig. 1B and Supplementary Fig. S4). They all have helicase activities, but *DDX58* and *DHX58* bind to DNA, and *IFIH1* and *FANCM* have RNA binding affinities. Functionally, they could participate in RISC-like complexes and respond to exogenous stress. *RNC* encodes dsRNA-specific RNase III.

There are four DICERs (*DCL1-4*) in plants^38^. Supplementary Fig. S4 shows a monophyletic group of plant DICERs containing four subclades: *DCL1*, *DCL2*, *DCL3* and *DCL4*. Based on the maturation types of plant small RNAs, these DICERs were grouped into two classes: (I) multifunctional DICERs (*DCL1*); and (II) siRNA-associated DICERs (*DCL2-4*). *DCL1* participates in RISC formation to process miRNA/siRNA precursors. *AtDCL2–4* generate siRNAs and are implicated in virus defense and production of siRNAs from natural *cis*-acting antisense transcripts, chromatin modification guidance or vegetative phase change regulation^39–41^. *RTL3* (*O. sativa*) belongs to the *DCL1* subclade and the RNase III family, suggesting that it possibly involves the miRNA/siRNA pathway. Furthermore, more DICER isoforms were found in *O. sativa*, indicating that a gene duplication event might have occurred during rice DICER evolution. A plant clade locates at outgroup, including *RTL3* (*A. thaliana*) and *RTL2* (*A. thaliana* and *O. sativa*), which are ribonucleases cleaving dsRNA and producing small RNAs.

Fungi (Ascomycota) Dicers show two subclades supported by a bootstrap value of 64: (I) *DCL1, DCR1*(*Schizosaccharomyces pombe*, *S. pombe*); and (II) *DCL2* (Fig. 1B and Supplementary Fig. S4). In vegetative cells, *DCL2* is a major Dicer enzyme in the process of siRNA biogenesis, but *DCL1* has a redundant role^42^. However, only *DCL1* is specifically expressed and required for meiotic silencing during meiosis^43^. In the siRNA maturation process, dsRNA precursors are processed by *DCR1* with similar domains of canonical DICER in animals. *DCR1* (*S. pombe*) is a Dicer homologue in fission yeast. *MPH1* is an ATP-dependent DNA helicase associated with DNA damage response to maintain genome integrity^44^. Yeast *MFH2* has DNA binding and DNA helicase activities. Other clades are mainly from bacterial *RNC*s (Actinobacteria, Aquificae, Firmicutes, Proteobacteria, Tenericutes and Thermotogae) which encode RNase and cleave RNAs (Fig. 1B and Supplementary Fig. S4).

### Evolution of RISC-related TRBP and PACT members

TRBP is implicated in *HIV-1* gene expression^45^, possibly linking miRNAs and the response of the IFN-PKR pathway to HIV-1 infection^46^. In vertebrates, TRBP is a paralogue to the protein kinase R (PKR)-activating protein or PACT^46,47^. They regulate PKR as an inhibitor (TRBP) or activator (PACT). RISC-related biological functions of TRBP and/or PACT with three DRSM domains (which bind to dsRNAs and mediate protein-protein interaction), shown in Supplementary Fig. S2, are the following: recruiting substrates to Dicer, facilitating Dicer-mediated processing of immature miRNAs, removing the Dicer product and controlling which type of dsRNA is loaded onto AGOs^12,13^.

In our phylogenetic trees, *TRBP*s*/PACT*s are shown only in Chordata (Fig. 1C,D and Supplementary Figs S5 and S6). Otherwise, *Drosophila R2D2* and *C. elegans RDE-4*, both known participants in RNAi processing, are distantly related to *TRBP/PACT* and do not emerge in chordate clades of *TRBP/PACT*. Loquacious has been identified in insects, but they are not considered because of low sequence identity and unreviewed entry in UniProt (Fig. 1C,D). Other evolutionarily related RNA/DNA binding genes are *DSRAD* (Chordata), *STAU1/2/STAUH* (Chordata, Mollusca and Arthropoda), *ILF3* (Chordata), *STRBP* (Chordata), *RED1/2* (Chordata) and *RNC* (Chloroflexi and Proteobacteria). *DSRAD*s are involved in RNA editing and facilitate loading of miRNA onto RISC^48^. Chordate *STAU1/2/STAUH* seems to function in mRNA transport or distribution^49,50^, and similar to miRNA precursors, Exportin-5 transports Staufen-dsRNA complexes out of the nucleus^51^. Chordate *ILF3* binds to RNA and functions in the biogenesis of circular RNAs (circRNAs). Chordate *STRBP* regulates spermatogenesis and sperm function through binding dsDNA/RNA. Chordate *RED1* catalyzes A-to-I RNA editing to affect gene expression and function. Chordate *RED2* with adenosine deaminase activity and dsRNA/ssRNA binding affinity prevents the binding of other *ADAR* enzymes to targets and reduces the efficiency of these enzymes. *RNC*s in Chloroflexi and Proteobacteria belong to RNase III family. Unexpectedly, *DRB2* (*O. sativa*) and *IIV6-340R* (*Invertebrate iridescent virus 6*) were located at the clades of chordate *DSRAD* and bacterial *RNC*s in our phylogenetic tree of *PACT*, respectively (Fig. 1D and Supplementary Fig. S6). They possibly bind to and cleave RNAs in plants or their host, but their RISC-related functions are still unknown.

### Evolution of RISC-related GW182 members

*GW182* identified in animals is a key component of RISC, interacts PIWI domain of *AGO1* via its N-terminal region for miRNA-mediated gene regulation (Supplementary Fig. S1) and exhibits high diversities in sequence length, conservation and composition. In this study, the full-length sequences of *GW182* and its orthologues were used for a reliable phylogenetic reconstruction, which was consistent with previous results (Supplementary Fig. S7)^17^. The results indicate that mammalian *TNRC6C* is the founding member of the chordate gene family representing and diverging from the orthologue of nonchordate *GW182* genes.

In our study, a limited number of *GW182* were identified and characterized, including human, mouse and fly (Fig. 1E and Supplementary Fig. S7). A further database BLAST search analysis of *GW182* mRNA/protein showed no additional homologues with highly convinced sequence similarities and biological functions, even in Nematoda. Instead, two functional analogues, *AIN-1* and *AIN-2*, are encoded in the genome of *C. elegans*. The observation is not surprising because more than half of the genes encoded in Nematoda are unique^52^.

### Gene duplication analysis of the major RISC members

Gene duplication is a crucial driving force of phenotype diversity, the cause of human diseases, and evolution. In our study, we quantified gene duplication events of the major RISC members. There were 51 gene duplications identified in the tree of AGOs (Supplementary Fig. S8). Class I, II and III of animal AGOs contained 6, 4 and 14 gene duplications, respectively. Class I, II and III of plant AGOs included 13, 3 and 5 gene duplications, respectively. In the tree of DICERs, 61 gene duplications were determined (Supplementary Fig. S9). Of this class of animal DICERs, 4 gene duplications arose from Chordata. In the two classes of plant DICERs, only class II had 6 gene duplications. For TRBP/PACT/GW182, there were 68/27/10 gene duplications, in which the numbers of their closely associated gene duplications were 1, 3 and 2, respectively (Supplementary Figs 10–12). These results suggest that plenty of gene duplications in AGOs and DICERs may be the main contributor to evolutionary diversity.

## Discussion

RISC-mediated gene regulation is vital for growth, development and metabolic disorders such as mitochondrial uncoupling proteins-mediated obesity and diabetes^53–55^. RISC is a large protein complex, in which its composition and protein copy numbers apparently affect its function. Previous investigations have demonstrated that RISCs originating from different species have distinct compositions; this phenomenon may mainly result from evolutionary selection pressures and the complexity of RISCs. To explore RISC evolution, phylogenetic trees and gene duplications of the major RISC members (AGOs, DICERs, TRBPs, PACTs and GW182s) were analyzed. These RISC components have various distributions in animals, plants, protista and fungi. TRBP/PACT/GW182 were observed only in animals. The monophyletic groups in the phylogenetic trees were as follows: mammalian AGOs, animal DICERs, chordate TRBP/PACT. RISC compositions in Arthropoda and Nematoda showed the evolutionary complexities, possibly derived from their unique functions.

Recently, Niels Wynant *et al*. have investigated the evolution of animal AGOs and reported three conserved AGO functional lineages: siRNA-class AGO, miRNA-class AGO and PIWI AGOs^15^. Here, we provide a refined classification of animal AGOs: multifunctional AGOs, siRNA-associated AGOs and piRNA-associated AGOs. Also, Fig. 1A points out a group of species- and sequence identity-based classes: Chordata (3 subclasses: *AGO2*, *AGO1/3/4* and *PIWIL1–4*), Arthropoda (2 subclasses: *AGO*2, *AGO3/AUB/PIWI/SIWI*), Nematoda (2 subclasses: *TAG-76*, *ERGO1/WAGO1/YQ53/NRDE3*) and Platyhelminthes. Homologous prokaryotic AGO proteins were discovered, but their functions are elusive because Archaea and Bacteria are deficiency of RNAi pathway^56^.

MiRNAs are identified in *Giardia intestinalis* (*G. intestinalis*), and they all target the open reading frame, but not the classic 3′-*UTR*s because their 3′-*UTR*s of genes are too short^57^. Therefore, *G. intestinalis* may generate a special RISC to interact with the miRNAs.

DICERs belong to the RNase III family which recognizes dsRNAs and cleaves them at specific targeted locations. In this study, many RNase IIIs were identified and investigated from eukaryotes to prokaryotes. Based on the structural differences of RNase IIIs, they were grouped into four classes: class 1 (bacteria and bacteriophage), class 2 (fungi), class 3 and class 4. DICER is classified as class 4 RNase III because it possesses both helicase and PAZ domains (Supplementary Fig. S2). A PAZ domain and two RNase III domains from *G. intestinalis* have been discovered by X-ray crystallography^58^.

Like function-dependent animal AGOs, plant *AGO/DICER* families have diversified extensively and appeared to possess plant-specific *AGO*s/*DICER*s. The identification of the 5′ terminal nucleotide of plant small RNA and miRNA-duplex structure strongly affect the loading of small RNAs onto specific *AGO*s. *DCL1* processes miRNA, *DCL1–4* process hairpin-derived siRNAs, and natural antisense siRNAs are processed by *DCL1, DCL2* or *DCL3*. The precursors of secondary siRNA transcribed by Pol II are trimmed by *DCL2/DCL4* to be siRNA. Heterochromatic siRNAs maturation is mediated by *DCL3*. It is likely that the diversification and specialization of RISC compositions in plants probably represent their roles in adaptation to a sessile lifestyle.

Yeast is miRNA pathway-deficient, but in fission yeast, AGO, DICER and RNA-dependent RNA polymerase factors are identified and used for the RNAi pathway, which are valuable for heterochromatin formation at the centromeres and mating type region. Therefore, fission yeast could be a model for studying the RNAi pathway. Moreover, the synthesis of dsRNAs is mediated by RDRs. The three major clades of eukaryotic RDRs are RDRα, RDRβ and RDRγ. In the fission yeast *S. pombe*, RDRγ is implicated in transcriptional gene silencing. These results indicate that yeast AGO/DICER/RDRs evolution may be integrated.

The related genes of chordate TRBP/PACT shown in Fig. 1C,D are mostly able to bind to RNA/DNA and have a variety of functions, such as RNA editing, mRNA transport, circRNAs biogenesis and RNA cleavage. These findings indicate that TRBP/PACT possibly perform the biological functions of their evolutionarily related genes in RISC besides the substrates recruitment and loading.

Gene duplication acts as an evolutionary engine so that it constitute a necessary force for evolutionary innovation and provide new genetic materials for new genes. A series of gene duplication events were found in RISC members, including AGOs and DICERs, which mostly existed in higher organisms. Gene duplications may be produced by unequal crossingover, retrotransposition, duplicated DNA transposition and polyploidization, but the molecular mechanisms of gene duplication in the major RISC members still remain ambiguous. Sometimes, gene duplications result in functional redundancies, such as plant complementary functioning AGOs as classified in our study. To minimize the genome sizes, CRISPR system has been widely used for gene editing^59,60^. The current results may provide assistance in improving or minimizing RISC and the genomes for achieving better efficiency and accuracy.

In summary, we systematically analyzed the evolution of the major RISC members, including AGO, DICER, TRBP, PACT and GW182. The findings provide potential support of species-specific RNAi/miRNA related RISC system optimization and model organism development.

## Methods

### Protein (amino acid) sequences retrievals

Key protein sequences of the major RISC members (AGO2 *Homo sapiens*-Q9UKV8, DICER *Homo sapiens*- Q9UPY3, TRBP2 *Homo sapiens*- Q15633, PRKRA *Homo sapiens*- O75569, GAWKY *Drosophila melanogaster*- Q8SY33) as the queries are used for searching all RISC-related members via BLAST algorithm, and subsequently, all reviewed protein sequences were retrieved from UniProt^61,62^. Homology cut-offs were E-values ≤0.0001 with the consideration of BLAST scores^62^. All detailed protein/gene information was listed in Supplementary Tables S1–5.

### Multiple sequence alignments

There are 93 (AGOs), 205 (DICERs), 124 (TRBPs) 57 (PACTs) and 15 (GW182s) protein entries used for multiple sequence alignments. Initial multiple sequence alignments were performed using the program MUSCLE (MUltiple Sequence Comparison by Log- Expectation) for reaching better average accuracy and speed^63^, The default settings (Gap Open:−2.9, Hydrophobicity Multiplier: 1.2, Clustering Methods: UPGMB, Min Diag Length: 24.) in MUSCLE were chosen for best accuracy and speed.

### Phylogenetic analysis

Several methods in MEGA7 are capable of constructing phylogenetic trees. In the present study, similar results were obtained from these methods, so the phylogenetic tree constructing by Neighbor-joining method was shown^64,65^. The percentage of replicate trees evaluated by booststrap test (1,000 replicates) were labeled next to the branches^66^. Possion correction method computed their evolutionary distances which were in the units of the number of amino acid substitutions per site.

### Domain architecture analysis

SMART (a Simple Modular Architecture Research Tool) was applied for identifying, annotating and analyzing domain architecture of the major RISC members^67^. Protein sequences obtained from UniProt were input into SMART server which searched Pfam domains of all proteins of interest.

### Finding gene duplications

Gene duplications were analyzed by “Find gene duplications” module in MEGA7^64,68^. Gene duplications were identified by searching for all branching points in the topology with at least one species present in both subtrees of the branching point. An unrooted gene tree was used for the analysis such that the search for duplication events was performed by finding the placement of the root on a branch or branches that produced the minimum number of duplication events.

## Electronic supplementary material

Supporting Information (Table S1–5 and Figure S1–12)
