# Supplementary material for: Comprehensive Evolutionary Analysis of the Major RNA-Induced Silencing Complex Members
Source: Sci Rep. 2018 Sep 21;8:14189. doi: 10.1038/s41598-018-32635-4 (PMC6155107; doi:10.1038/s41598-018-32635-4)
Supplement: Supplementary file 1 — Supporting Information (Table S1–5 and Figure S1–12) [file 41598_2018_32635_MOESM1_ESM.pdf]

Electronic Supporting Information

Comprehensive Evolutionary Analysis of the Major RNA-Induced Silencing Complex Members

Rui Zhang[1,a], Ying Jing[1,a], Haiyang Zhang[1], Yahan Niu[1], Chang Liu[1], Jin Wang[1], Ke Zen[1], Chen-Yu Zhang[1], Donghai Li[1,*]

[1]*State Key Laboratory of Pharmaceutical Biotechnology, Jiangsu Engineering Research Center for MicroRNA Biology and Biotechnology, Nanjing Advanced Institute for Life Sciences (NAILS), School of Life Sciences, Nanjing University, Nanjing, Jiangsu 210023, P.R. China*

[a]*Contributed equally*

[*]*Correspondence to:*

Donghai Li

State Key Laboratory of Pharmaceutical Biotechnology, Jiangsu Engineering Research Center for MicroRNA Biology and Biotechnology, Nanjing Advanced Institute for Life Sciences (NAILS), School of Life Sciences, Nanjing University, Nanjing, Jiangsu 210023, P.R. China

E-mail: donghaili@nju.edu.cn

Supporting Information

Table S1. Detailed sequence information of AGO members used for constructing a phylogenetic tree.

Entry	Gene names	Taxonomic lineage (SPECIES)	Taxonomic lineage	Score	Identity	Protein names
**Q9UKV8**	*AGO2*	*Homo sapiens (Human)*	Chordata	4,563	100.00%	Protein argonaute-2 (Argonaute2) (hAgo2) (EC 3.1.26.n2) (Argonaute RISC catalytic component 2) (Eukaryotic translation initiation factor 2C 2) (eIF-2C 2) (eIF2C 2) (PAZ Piwi domain protein) (PPD) (Protein slicer)
**Q6QME8**	*AGO2*	*Bos taurus (Bovine)*	Chordata	4,540	99.80%	Protein argonaute-2 (Argonaute2) (EC 3.1.26.n2) (Argonaute RISC catalytic component 2) (Eukaryotic translation initiation factor 2C 2) (eIF-2C 2) (eIF2C 2) (Protein slicer)
**Q8CJG0**	*AGO2*	*Mus musculus (Mouse)*	Chordata	4,514	99.20%	Protein argonaute-2 (Argonaute2) (mAgo2) (EC 3.1.26.n2) (Argonaute RISC catalytic component 2) (Eukaryotic translation initiation factor 2C 2) (eIF-2C 2) (eIF2C 2) (Piwi/argonaute family protein meIF2C2) (Protein slicer)
**Q9QZ81**	*AGO2*	*Rattus norvegicus (Rat)*	Chordata	4,510	99.10%	Protein argonaute-2 (Argonaute2) (EC 3.1.26.n2) (Argonaute RISC catalytic component 2) (Eukaryotic translation initiation factor 2C 2) (eIF-2C 2) (eIF2C 2) (Golgi ER protein 95 kDa) (GERp95) (Protein slicer)
**O77503**	*AGO2*	*Oryctolagus cuniculus (Rabbit)*	Chordata	4,441	99.60%	Protein argonaute-2 (Argonaute2) (EC 3.1.26.n2) (Argonaute RISC catalytic component 2) (Eukaryotic translation initiation factor 2C 2) (eIF-2C 2) (eIF2C 2) (Protein slicer) (Fragment)
**Q6DCX2**	*AGO2*	*Xenopus laevis (African clawed frog)*	Chordata	4,414	96.20%	Protein argonaute-2 (Argonaute2) (EC 3.1.26.n2) (Argonaute RISC catalytic component 2) (Eukaryotic translation initiation factor 2C 2) (eIF-2C 2) (eIF2C 2) (Protein slicer)
**Q6DJB9**	*AGO2*	*Xenopus tropicalis (Western clawed frog) (Silurana tropicalis)*	Chordata	4,413	95.60%	Protein argonaute-2 (Argonaute2) (EC 3.1.26.n2) (Argonaute RISC catalytic component 2) (Eukaryotic translation initiation factor 2C 2) (eIF-2C 2) (eIF2C 2) (Protein slicer)
**Q9UKV8-2**	*AGO2*	*Homo sapiens (Human)*	Chordata	4,333	96.00%	Isoform 2 of Protein argonaute-2
**Q9UL18**	*AGO1*	*Homo sapiens (Human)*	Chordata	3,814	83.00%	Protein argonaute-1 (Argonaute1) (hAgo1) (Argonaute RISC catalytic component 1) (Eukaryotic translation initiation factor 2C 1) (eIF-2C 1) (eIF2C 1) (Putative RNA-binding protein Q99)
**Q8CJG1**	*AGO1*	*Mus musculus (Mouse)*	Chordata	3,814	83.00%	Protein argonaute-1 (Argonaute1) (mAgo1) (Argonaute RISC catalytic component 1) (Eukaryotic translation initiation factor 2C 1) (eIF-2C 1) (eIF2C 1) (Piwi/argonaute family protein meIF2C1)
**Q9H9G7**	*AGO3*	*Homo sapiens (Human)*	Chordata	3,672	79.70%	Protein argonaute-3 (Argonaute3) (hAgo3) (Argonaute RISC catalytic component 3) (Eukaryotic translation initiation factor 2C 3) (eIF-2C 3) (eIF2C 3)
**Q8CJF9**	*AGO3*	*Mus musculus (Mouse)*	Chordata	3,667	79.60%	Protein argonaute-3 (Argonaute3) (mAgo3) (Argonaute RISC catalytic component 3) (Eukaryotic translation initiation factor 2C 3) (eIF-2C 3) (eIF2C 3) (Piwi/argonaute family protein meIF2C3)
**A3KPK0**	*AGO3*	*Danio rerio (Zebrafish) (Brachydanio rerio)*	Chordata	3,661	80.10%	Protein argonaute-3 (Argonaute3) (Argonaute RISC catalytic component 3) (Eukaryotic translation initiation factor 2C 3) (eIF-2C 3) (eIF2C 3)
**Q5ZLG4**	*AGO3*	*Gallus gallus (Chicken)*	Chordata	3,659	79.30%	Protein argonaute-3 (Argonaute3) (Argonaute RISC catalytic component 3) (Eukaryotic translation initiation factor 2C 3) (eIF-2C 3) (eIF2C 3)

**Q9HCK5**	*AGO4*	*Homo sapiens (Human)*	Chordata	3,628	78.40%	Protein argonaute-4 (Argonaute4) (hAgo4) (Argonaute RISC catalytic component 4) (Eukaryotic translation initiation factor 2C 4) (eIF-2C 4) (eIF2C 4)
**Q4KLV6**	*AGO4*	*Xenopus laevis (African clawed frog)*	Chordata	3,618	77.70%	Protein argonaute-4 (Argonaute4) (Argonaute RISC catalytic component 4) (Eukaryotic translation initiation factor 2C 4) (eIF-2C 4) (eIF2C 4)
**Q8CJF8**	*AGO4*	*Mus musculus (Mouse)*	Chordata	3,615	77.70%	Protein argonaute-4 (Argonaute4) (mAgo4) (Argonaute RISC catalytic component 4) (Eukaryotic translation initiation factor 2C 4) (eIF-2C 4) (eIF2C 4) (Piwi/argonaute family protein meIF2C4)
**Q6T5B7**	*AGO3*	*Bos taurus (Bovine)*	Chordata	3,604	79.90%	Protein argonaute-3 (Argonaute3) (Argonaute RISC catalytic component 3) (Eukaryotic translation initiation factor 2C 3) (eIF-2C 3) (eIF2C 3)
**Q5ZMW0**	*AGO4*	*Gallus gallus (Chicken)*	Chordata	3,422	80.10%	Protein argonaute-4 (Argonaute4) (Argonaute RISC catalytic component 4) (Eukaryotic translation initiation factor 2C 4) (eIF-2C 4) (eIF2C 4)
**Q8CJF8-2**	*AGO4*	*Mus musculus (Mouse)*	Chordata	2,937	66.90%	Isoform 2 of Protein argonaute-4
**Q9H9G7-2**	*AGO3*	*Homo sapiens (Human)*	Chordata	2,895	85.60%	Isoform 2 of Protein argonaute-3
**Q69VD5**	*PHN1*	*Oryza sativa (Rice)*	Streptophyta	1,753	41.50%	Protein argonaute PNH1 (Protein PINHEAD homolog 1) (OsPNH1)
**O04379**	*AGO1*	*Arabidopsis thaliana (Mouse-ear cress)*	Streptophyta	1,745	44.00%	Protein argonaute 1
**Q6K972**	*AGO1C*	*Oryza sativa (Rice)*	Streptophyta	1,736	44.00%	Protein argonaute 1C (OsAGO1c) (Protein argonaute 1) (OsAGO1)
**O04379-2**	*AGO1*	*Arabidopsis thaliana (Mouse-ear cress)*	Streptophyta	1,734	43.90%	Isoform 2 of Protein argonaute 1
**Q5Z5B2**	*AGO1D*	*Oryza sativa (Rice)*	Streptophyta	1,727	43.60%	Protein argonaute 1D (OsAGO1d)
**Q6EU14**	*AGO1A*	*Oryza sativa (Rice)*	Streptophyta	1,717	43.60%	Protein argonaute 1A (OsAGO1a)
**Q7XSA2-2**	*AGO1B*	*Oryza sativa (Rice)*	Streptophyta	1,713	43.80%	Isoform 2 of Protein argonaute 1B
**Q7XSA2**	*AGO1B*	*Oryza sativa (Rice)*	Streptophyta	1,713	43.80%	Protein argonaute 1B (OsAGO1b)
**Q9XGW1**	*AGO10*	*Arabidopsis thaliana (Mouse-ear cress)*	Streptophyta	1,699	42.80%	Protein argonaute 10 (Protein PINHEAD) (Protein ZWILLE)
**Q851R2**	*MEL1*	*Oryza sativa (Rice)*	Streptophyta	1,659	41.30%	Protein argonaute MEL1 (Protein MEIOSIS ARRESTED AT LEPTOTENE 1) (OsMEL1)
**Q7Y001**	*AGO12*	*Oryza sativa (Rice)*	Streptophyta	1,638	41.50%	Protein argonaute 12 (OsAGO12)
**Q9SJK3**	*AGO5*	*Arabidopsis thaliana (Mouse-ear cress)*	Streptophyta	1,595	40.70%	Protein argonaute 5
**Q6Z4F1**	*AGO14*	*Oryza sativa (Rice)*	Streptophyta	1,494	39.50%	Protein argonaute 14 (OsAGO14)
**Q69UP6**	*AGO18*	*Oryza sativa (Rice)*	Streptophyta	1,343	39.80%	Protein argonaute 18 (OsAGO18)
**Q852N2**	*AGO13*	*Oryza sativa (Rice)*	Streptophyta	1,319	37.80%	Protein argonaute 13 (OsAGO13)
**Q9C793**	*AGO7*	*Arabidopsis thaliana (Mouse-ear cress)*	Streptophyta	1,255	37.40%	Protein argonaute 7 (Protein ZIPPY)

**Q10F39**	*AGO11*	*Oryza sativa (Rice)*	Streptophyta	1,249	36.00%	Protein argonaute 11 (OsAGO11)
**Q75HC2**	*AGO7*	*Oryza sativa (Rice)*	Streptophyta	1,244	37.10%	Protein argonaute 7 (OsAGO7) (Protein SHOOT ORGANIZATION 2) (Protein SHOOTLESS 4)
**P34681-2**	*TAG-76*	*Caenorhabditis elegans*	Nematoda	1,185	42.60%	Isoform b of Putative protein tag-76
**P34681**	*TAG-76*	*Caenorhabditis elegans*	Nematoda	1,185	42.60%	Putative protein tag-76
**Q7XTS4**	*AGO2*	*Oryza sativa (Rice)*	Streptophyta	1,141	34.00%	Protein argonaute 2 (OsAGO2)
**Q6H6C3**	*AGO17*	*Oryza sativa (Rice)*	Streptophyta	1,138	33.70%	Protein argonaute 17 (OsAGO17)
**O74957**	*AGO1*	*Schizosaccharomyces pombe (Fission yeast)*	Ascomycota	1,103	33.50%	Protein argonaute (Cell cycle control protein ago1) (Eukaryotic translation initiation factor 2C 2-like protein ago1) (PAZ Piwi domain protein ago1) (Protein slicer) (RNA interference pathway protein ago1)
**Q9ZVD5**	*AGO4*	*Arabidopsis thaliana (Mouse-ear cress)*	Streptophyta	1,082	32.30%	Protein argonaute 4 (Protein OVEREXPRESSOR OF CATIONIC PEROXIDASE 11)
**Q9SHF3**	*AGO2*	*Arabidopsis thaliana (Mouse-ear cress)*	Streptophyta	1,018	31.60%	Protein argonaute 2
**Q0JF58**	*AGO4B*	*Oryza sativa (Rice)*	Streptophyta	1,004	31.00%	Protein argonaute 4B (OsAGO4b)
**Q6YSJ5**	*AGO16*	*Oryza sativa (Rice)*	Streptophyta	993	31.60%	Protein argonaute 16 (OsAGO16) (OsAGO6) (Protein ZWILLE/PINHEAD-like 1)
**Q9SHF2**	*AGO3*	*Arabidopsis thaliana (Mouse-ear cress)*	Streptophyta	984	31.40%	Protein argonaute 3
**Q84VQ0**	*AGO9*	*Arabidopsis thaliana (Mouse-ear cress)*	Streptophyta	981	30.50%	Protein argonaute 9
**Q7XTS3**	*AGO3*	*Oryza sativa (Rice)*	Streptophyta	979	32.50%	Protein argonaute 3 (OsAGO3)
**O48771**	*AGO6*	*Arabidopsis thaliana (Mouse-ear cress)*	Streptophyta	968	32.70%	Protein argonaute 6
**Q9SDG8**	*AGO4A*	*Oryza sativa (Rice)*	Streptophyta	963	30.10%	Protein argonaute 4A (OsAGO4a)
**Q9VUQ5**	*AGO2*	*Drosophila melanogaster (Fruit fly)*	Arthropoda	948	31.70%	Protein argonaute-2
**Q9VUQ5-2**	*AGO2*	*Drosophila melanogaster (Fruit fly)*	Arthropoda	948	31.70%	Isoform C of Protein argonaute-2
**Q5NBN9**	*AGO15*	*Oryza sativa (Rice)*	Streptophyta	929	30.90%	Protein argonaute 15 (OsAGO15)
**Q3E984**	*AGO8*	*Arabidopsis thaliana (Mouse-ear cress)*	Streptophyta	815	30.30%	Protein argonaute 8
**O61931**	*ERGO1*	*Caenorhabditis elegans*	Nematoda	530	28.50%	Piwi-like protein ergo-1 (Endogenous RNA interference deficient argonaute protein 1)
**A6N7Y9**	*PIWIL1*	*Gallus gallus (Chicken)*	Chordata	501	25.40%	Piwi-like protein 1 (EC 3.1.26.-)
**Q8UVX0**	*PIWIL1*	*Danio rerio (Zebrafish) (Brachydanio rerio)*	Chordata	488	26.30%	Piwi-like protein 1 (EC 3.1.26.-)

**A9ZSZ2**	*AGO3*	*Bombyx mori (Silk moth)*	Arthropoda	466	24.60%	Piwi-like protein Ago3 (BmAGO3) (EC 3.1.26.-)
**Q8CGT6**	*PIWIL4*	*Mus musculus (Mouse)*	Chordata	448	24.30%	Piwi-like protein 4 (mAgo5)
**Q4G033**	*PIWIL4*	*Rattus norvegicus (Rat)*	Chordata	448	24.20%	Piwi-like protein 4
**A8D8P8**	*SIWI*	*Bombyx mori (Silk moth)*	Arthropoda	447	24.10%	Piwi-like protein Siwi (EC 3.1.26.-)
**Q8CGT6-3**	*PIWIL4*	*Mus musculus (Mouse)*	Chordata	445	24.50%	Isoform 3 of Piwi-like protein 4
**Q96J94-3**	*PIWIL1*	*Homo sapiens (Human)*	Chordata	426	24.10%	Isoform 3 of Piwi-like protein 1
**Q96J94**	*PIWIL1*	*Homo sapiens (Human)*	Chordata	426	24.10%	Piwi-like protein 1 (EC 3.1.26.-)
**Q7Z3Z4**	*PIWIL4*	*Homo sapiens (Human)*	Chordata	425	23.90%	Piwi-like protein 4
**Q9JMB7**	*PIWIL1*	*Mus musculus (Mouse)*	Chordata	413	23.50%	Piwi-like protein 1 (EC 3.1.26.-)
**Q8CGT6-2**	*PIWIL4*	*Mus musculus (Mouse)*	Chordata	405	24.00%	Isoform 2 of Piwi-like protein 4
**Q7Z3Z3**	*PIWIL3*	*Homo sapiens (Human)*	Chordata	395	24.60%	Piwi-like protein 3
**Q96J94-2**	*PIWIL1*	*Homo sapiens (Human)*	Chordata	385	23.80%	Isoform 2 of Piwi-like protein 1
**O76922-2**	*AUB*	*Drosophila melanogaster (Fruit fly)*	Arthropoda	378	22.50%	Isoform C of Protein aubergine
**O76922**	*AUB*	*Drosophila melanogaster (Fruit fly)*	Arthropoda	378	22.50%	Protein aubergine (Protein sting)
**Q21770**	*WAGO1*	*Caenorhabditis elegans*	Nematoda	377	26.30%	Argonaute protein wago-1 (Worm-specific argonaute protein 1)
**Q8TC59**	*PIWIL2*	*Homo sapiens (Human)*	Chordata	374	23.20%	Piwi-like protein 2 (EC 3.1.26.-) (Cancer/testis antigen 80) (CT80)
**Q9JMB7-2**	*PIWIL1*	*Mus musculus (Mouse)*	Chordata	372	23.20%	Isoform 2 of Piwi-like protein 1
**A2CEI6**	*PIWIL2*	*Danio rerio (Zebrafish) (Brachydanio rerio)*	Chordata	370	22.70%	Piwi-like protein 2 (EC 3.1.26.-)
**Q9VKM1**	*PIWI*	*Drosophila melanogaster (Fruit fly)*	Arthropoda	368	23.40%	Protein piwi (EC 3.1.26.-)
**A8KBF3**	*PIWIL2*	*Xenopus tropicalis (Western clawed frog) (Silurana tropicalis)*	Chordata	366	22.90%	Piwi-like protein 2 (EC 3.1.26.-)
**Q09249**	*YQ53, C16C10.3*	*Caenorhabditis elegans*	Nematoda	362	24.70%	Uncharacterized protein C16C10.3
**Q7PLK0**	*AGO3*	*Drosophila melanogaster (Fruit fly)*	Arthropoda	360	24.60%	Protein argonaute-3
**Q8CDG1**	*PIWIL2*	*Mus musculus (Mouse)*	Chordata	355	22.60%	Piwi-like protein 2 (EC 3.1.26.-)
**Q7PLK0-2**	*AGO3*	*Drosophila melanogaster (Fruit fly)*	Arthropoda	348	24.30%	Isoform G of Protein argonaute-3

**A6P7L8**	*PIWIL2*	*Oncorhynchus mykiss (Rainbow trout) (Salmo gairdneri)*	Chordata	346	22.90%	Piwi-like protein 2 (EC 3.1.26.-)
**Q21691**	*NRDE3*	*Caenorhabditis elegans*	Nematoda	328	22.80%	Nuclear RNAi defective-3 protein
**Q2PC95**	*PIWIL*	*Dugesia japonica (Planarian)*	Platyhelminthes	308	24.70%	Piwi-like protein
**Q2Q5Y9**	*PIWI1*	*Schmidtea mediterranea (Freshwater planarian flatworm)*	Platyhelminthes	278	25.90%	Piwi-like protein 1 (SMEDWI-1)
**Q8TC59-2**	*PIWIL2*	*Homo sapiens (Human)*	Chordata	275	21.60%	Isoform 2 of Piwi-like protein 2
**Q2Q5Y8**	*PIWI2*	*Schmidtea mediterranea (Freshwater planarian flatworm)*	Platyhelminthes	266	25.60%	Piwi-like protein 2 (SMEDWI-2)
**C6LTG5**	*AGO*	*Giardia intestinalis (Giardia lamblia)*	Sarcomastigophoraa	190	27.20%	Protein argonaute
**Q86QW7**	*AGO*	*Giardia intestinalis (Giardia lamblia)*	Sarcomastigophoraa	167	22.00%	Protein argonaute (GlAgo)
**A8BCK6**	*AGO*	*Giardia intestinalis (Giardia lamblia)*	Sarcomastigophoraa	167	22.00%	Protein argonaute

Table S2.Detailed sequence information of DICER members used for constructing a phylogenetic tree.

Entry	Gene names	Taxonomic lineage (SPECIES)	Taxonomic lineage	Score	Identity	Protein names
**Q9UPY3**	*DICER1*	*Homo sapiens (Human)*	Chordata	10,119	100.00%	Endoribonuclease Dicer (EC 3.1.26.3) (Helicase with RNase motif) (Helicase MOI)
**Q6TUI4**	*DICER1*	*Bos taurus (Bovine)*	Chordata	9,682	95.80%	Endoribonuclease Dicer (EC 3.1.26.3)
**A0MQH0**	*DICER1*	*Cricetulus griseus (Chinese hamster) (Cricetulus barabensis griseus)*	Chordata	9,557	94.40%	Endoribonuclease Dicer (EC 3.1.26.3)
**Q9UPY3-2**	*DICER1*	*Homo sapiens (Human)*	Chordata	9,443	100.00%	Isoform 2 of Endoribonuclease Dicer (Isoform t-Dicer of Endoribonuclease Dicer)
**Q8R418**	*DICER1*	*Mus musculus (Mouse)*	Chordata	9,407	92.90%	Endoribonuclease Dicer (EC 3.1.26.3) (Double-strand-specific ribonuclease mDCR-1)
**Q25BN1**	*DICER1*	*Gallus gallus (Chicken)*	Chordata	9,364	92.00%	Endoribonuclease Dicer (EC 3.1.26.3)
**B3DLA6**	*DICER1*	*Xenopus tropicalis (Western clawed frog) (Silurana tropicalis)*	Chordata	8,283	83.10%	Endoribonuclease Dicer (EC 3.1.26.3)
**Q8R418-2**	*DICER1*	*Mus musculus (Mouse)*	Chordata	8,201	92.70%	Isoform 2 of Endoribonuclease Dicer (Isoform DicerO of Endoribonuclease Dicer)
**Q6TV19**	*DICER1*	*Danio rerio (Zebrafish) (Brachydanio rerio)*	Chordata	7,810	79.20%	Endoribonuclease Dicer (EC 3.1.26.3)
**Q9UPY3-3**	*DICER1*	*Homo sapiens (Human)*	Chordata	4,257	100.00%	Isoform 3 of Endoribonuclease Dicer
**B3DLA6-2**	*DICER1*	*Xenopus tropicalis (Western clawed frog) (Silurana tropicalis)*	Chordata	3,836	89.20%	Isoform 2 of Endoribonuclease Dicer
**P34529**	*DCR1*	*Caenorhabditis elegans*	Nematoda	1,536	36.40%	Endoribonuclease dcr-1 (EC 3.1.26.-) [Cleaved into: Death-promoting deoxyribonuclease (tDCR-1) (EC 3.1.21.-)]
**Q9VCU9**	*DCR1*	*Drosophila melanogaster (Fruit fly)*	Arthropoda	1,091	43.50%	Endoribonuclease Dcr-1 (Protein dicer-1) (EC 3.1.26.-)
**Q9SP32**	*DCL1*	*Arabidopsis thaliana (Mouse-ear cress)*	Streptophyta	703	29.60%	Endoribonuclease Dicer homolog 1 (EC 3.1.26.-) (Dicer-like protein 1) (AtDCL1) (Protein ABNORMAL SUSPENSOR 1) (Protein CARPEL FACTORY) (Protein SHORT INTEGUMENTS 1) (Protein SUSPENSOR 1)
**Q8LMR2**	*DCL1*	*Oryza sativa (Rice)*	Streptophyta	637	29.30%	Endoribonuclease Dicer homolog 1 (Dicer-like protein 1) (OsDCL1) (EC 3.1.26.-)
**Q5N870**	*DCL3A*	*Oryza sativa (Rice)*	Streptophyta	621	29.90%	Endoribonuclease Dicer homolog 3a (Dicer-like protein 3a) (OsDCL3a) (EC 3.1.26.-)
**Q7XD96**	*DCL3B*	*Oryza sativa (Rice)*	Streptophyta	591	28.60%	Endoribonuclease Dicer homolog 3b (Dicer-like protein 3b) (OsDCL3b) (EC 3.1.26.-)
**Q1DKI1**	*DCL1*	*Coccidioides immitis (Valley fever fungus)*	Ascomycota	585	25.70%	Dicer-like protein 1 [Includes: Endoribonuclease DCL1 (EC 3.1.26.-); ATP-dependent helicase DCL1 (EC 3.6.4.-)]
**Q7S8J7**	*DCL1*	*Neurospora crassa*	Ascomycota	571	23.20%	Dicer-like protein 1 [Includes: Endoribonuclease dcl-1 (EC 3.1.26.-); ATP-dependent helicase dcl-1 (EC 3.6.4.-)]
**A4RKC3**	*DCL1*	*Magnaporthe oryzae (Rice blast fungus) (Pyricularia oryzae)*	Ascomycota	570	24.20%	Dicer-like protein 1 [Includes: Endoribonuclease DCL1 (EC 3.1.26.-); ATP-dependent helicase DCL1 (EC 3.6.4.-)]

**A7LFZ6**	*DCL4*	*Oryza sativa (Rice)*	Streptophyta	564	25.10%	Endoribonuclease Dicer homolog 4 (EC 3.1.26.-) (Dicer-like protein 4) (OsDCL4) (Protein SHOOT ORGANIZATION 1)
**Q10HL3**	*DCL2A*	*Oryza sativa (Rice)*	Streptophyta	553	27.00%	Endoribonuclease Dicer homolog 2a (Dicer-like protein 2a) (OsDCL2a) (EC 3.1.26.-)
**Q0UI93**	*DCL1*	*Phaeosphaeria nodorum (Glume blotch fungus) (Parastagonospora nodorum)*	Ascomycota	492	26.30%	Dicer-like protein 1 [Includes: Endoribonuclease DCL1 (EC 3.1.26.-); ATP-dependent helicase DCL1 (EC 3.6.4.-)]
**Q09884**	*DCR1*	*Schizosaccharomyces pombe (Fission yeast)*	Ascomycota	490	24.40%	Protein Dicer (Cell cycle control protein dcr1) (RNA interference pathway protein dcr1) [Includes: Endoribonuclease dcr1 (EC 3.1.26.-); ATP-dependent helicase dcr1 (EC 3.6.4.-)]
**Q2H0G2**	*DCL1*	*Chaetomium globosum (Soil fungus)*	Ascomycota	474	24.00%	Dicer-like protein 1 [Includes: Endoribonuclease DCL1 (EC 3.1.26.-); ATP-dependent helicase DCL1 (EC 3.6.4.-)]
**Q69LX2**	*DCL2B*	*Oryza sativa (Rice)*	Streptophyta	457	25.50%	Endoribonuclease Dicer homolog 2b (Dicer-like protein 2b) (OsDCL2b) (EC 3.1.26.-)
**Q2VF19**	*DCL1*	*Cryphonectria parasitica (Chestnut blight fungus) (Endothia parasitica)*	Ascomycota	456	23.70%	Dicer-like protein 1 [Includes: Endoribonuclease DCL-1 (EC 3.1.26.-); ATP-dependent helicase DCL-1 (EC 3.6.4.-)]
**Q2UNX5**	*DCL2*	*Aspergillus oryzae (Yellow koji mold)*	Ascomycota	408	24.50%	Dicer-like protein 2 [Includes: Endoribonuclease dcl2 (EC 3.1.26.-); ATP-dependent helicase dcl2 (EC 3.6.4.-)]
**Q2VF18**	*DCL2*	*Cryphonectria parasitica (Chestnut blight fungus) (Endothia parasitica)*	Ascomycota	405	24.40%	Dicer-like protein 2 [Includes: Endoribonuclease DCL-2 (EC 3.1.26.-); ATP-dependent helicase DCL-2 (EC 3.6.4.-)]
**Q4WA22**	*DCL2*	*Neosartorya fumigata (Aspergillus fumigatus)*	Ascomycota	394	24.70%	Dicer-like protein 2 [Includes: Endoribonuclease dcl2 (EC 3.1.26.-); ATP-dependent helicase dcl2 (EC 3.6.4.-)]
**Q3EBC8-2**	*DCL2*	*Arabidopsis thaliana (Mouse-ear cress)*	Streptophyta	383	32.70%	Isoform 2 of Endoribonuclease Dicer homolog 2
**Q3EBC8**	*DCL2*	*Arabidopsis thaliana (Mouse-ear cress)*	Streptophyta	383	32.70%	Endoribonuclease Dicer homolog 2 (EC 3.1.26.-) (Dicer-like protein 2) (AtDCL2)
**A1D9Z6**	*DCL2*	*Aspergillus fischeri*	Ascomycota	382	24.70%	Dicer-like protein 2 [Includes: Endoribonuclease dcl2 (EC 3.1.26.-); ATP-dependent helicase dcl2 (EC 3.6.4.-)]
**A7LFZ6-2**	*DCL4*	*Oryza sativa (Rice)*	Streptophyta	369	25.40%	Isoform 2 of Endoribonuclease Dicer homolog 4
**Q0UL22**	*DCL2*	*Phaeosphaeria nodorum (Glume blotch fungus) (Parastagonospora nodorum)*	Ascomycota	369	23.90%	Dicer-like protein 2 [Includes: Endoribonuclease DCL2 (EC 3.1.26.-); ATP-dependent helicase DCL2 (EC 3.6.4.-)]
**P0C5H7**	*DCL2*	*Emericella nidulans (Aspergillus nidulans)*	Ascomycota	365	26.00%	Dicer-like protein 2 [Includes: Endoribonuclease dcl2 (EC 3.1.26.-); ATP-dependent helicase dcl2 (EC 3.6.4.-)]
**Q9LXW7-2**	*DCL3*	*Arabidopsis thaliana (Mouse-ear cress)*	Streptophyta	350	29.60%	Isoform 2 of Endoribonuclease Dicer homolog 3
**Q9LXW7**	*DCL3*	*Arabidopsis thaliana (Mouse-ear cress)*	Streptophyta	350	29.60%	Endoribonuclease Dicer homolog 3 (EC 3.1.26.-) (Dicer-like protein 3) (AtDCL3)
**P84634**	*DCL4*	*Arabidopsis thaliana (Mouse-ear cress)*	Streptophyta	342	31.00%	Dicer-like protein 4 (EC 3.1.26.-)
**A1C9M6**	*DCL2*	*Aspergillus clavatus*	Ascomycota	339	24.10%	Dicer-like protein 2 [Includes: Endoribonuclease dcl2 (EC 3.1.26.-); ATP-dependent helicase dcl2 (EC 3.6.4.-)]
**Q0CW42**	*DCL1*	*Aspergillus terreus*	Ascomycota	333	34.30%	Dicer-like protein 1 [Includes: Endoribonuclease dcl1 (EC 3.1.26.-); ATP-dependent helicase dcl1 (EC

						3.6.4.-)]
**A2RAF3**	*DCL2*	*Aspergillus niger*	Ascomycota	330	37.40%	Dicer-like protein 1 [Includes: Endoribonuclease dcl1 (EC 3.1.26.-); ATP-dependent helicase dcl1 (EC 3.6.4.-)]
**Q1DW80**	*DCL2*	*Coccidioides immitis (Valley fever fungus)*	Ascomycota	321	25.20%	Dicer-like protein 2 [Includes: Endoribonuclease DCL2 (EC 3.1.26.-); ATP-dependent helicase DCL2 (EC 3.6.4.-)]
**Q2U6C4**	*DCL1*	*Aspergillus oryzae (Yellow koji mold)*	Ascomycota	312	37.20%	Dicer-like protein 1 [Includes: Endoribonuclease dcl1 (EC 3.1.26.-); ATP-dependent helicase dcl1 (EC 3.6.4.-)]
**A1DE13**	*DCL1*	*Aspergillus fischeri*	Ascomycota	311	34.60%	Dicer-like protein 1 [Includes: Endoribonuclease dcl1 (EC 3.1.26.-); ATP-dependent helicase dcl1 (EC 3.6.4.-)]
**Q4WVE3**	*DCL2*	*Neosartorya fumigata (Aspergillus fumigatus)*	Ascomycota	308	34.60%	Dicer-like protein 1 [Includes: Endoribonuclease dcl1 (EC 3.1.26.-); ATP-dependent helicase dcl1 (EC 3.6.4.-)]
**Q9GLV6**	*DDX58*	*Sus scrofa (Pig)*	Chordata	302	26.10%	Probable ATP-dependent RNA helicase DDX58 (EC 3.6.4.13) (DEAD box protein 58) (RHIV-1) (RIG-I-like receptor 1) (RLR-1) (RNA helicase induced by virus) (Retinoic acid-inducible gene 1 protein) (RIG-1) (Retinoic acid-inducible gene I protein) (RIG-I)
**A1CBC9**	*DCL1*	*Aspergillus clavatus*	Ascomycota	300	33.00%	Dicer-like protein 1 [Includes: Endoribonuclease dcl1 (EC 3.1.26.-); ATP-dependent helicase dcl1 (EC 3.6.4.-)]
**Q6Q899**	*DDX58*	*Mus musculus (Mouse)*	Chordata	297	24.30%	Probable ATP-dependent RNA helicase DDX58 (EC 3.6.4.13) (DEAD box protein 58) (RIG-I-like receptor 1) (RLR-1) (Retinoic acid-inducible gene 1 protein) (RIG-1) (Retinoic acid-inducible gene I protein) (RIG-I)
**O95786-2**	*DDX58*	*Homo sapiens (Human)*	Chordata	283	24.40%	Isoform 2 of Probable ATP-dependent RNA helicase DDX58
**O95786**	*DDX58*	*Homo sapiens (Human)*	Chordata	283	24.40%	Probable ATP-dependent RNA helicase DDX58 (EC 3.6.4.13) (DEAD box protein 58) (RIG-I-like receptor 1) (RLR-1) (Retinoic acid-inducible gene 1 protein) (RIG-1) (Retinoic acid-inducible gene I protein) (RIG-I)
**Q69KJ0**	*RTL3*	*Oryza sativa (Rice)*	Streptophyta	268	31.30%	Ribonuclease 3-like protein 3 (EC 3.1.26.-) (Ribonuclease III-like protein 3) (RNase III-like protein 3)
**A4RHU9**	*DCL2*	*Magnaporthe oryzae (Rice blast fungus) (Pyricularia oryzae)*	Ascomycota	264	27.80%	Dicer-like protein 2 [Includes: Endoribonuclease DCL2 (EC 3.1.26.-); ATP-dependent helicase DCL2 (EC 3.6.4.-)]
**A2R345**	*DCL2-1*	*Aspergillus niger*	Ascomycota	252	28.00%	Dicer-like protein 2-1 [Includes: Endoribonuclease dcl2-1 (EC 3.1.26.-); ATP-dependent helicase dcl2-1 (EC 3.6.4.-)]
**Q9LTQ0**	*RTL2*	*Arabidopsis thaliana (Mouse-ear cress)*	Streptophyta	248	27.80%	Ribonuclease 3-like protein 2 (EC 3.1.26.-) (Ribonuclease III-like protein 2) (RNase III-like protein 2) (Ribonuclease three-like protein 2)
**A2QX45**	*DCL2-2*	*Aspergillus niger*	Ascomycota	245	26.10%	Dicer-like protein 2-2 [Includes: Endoribonuclease dcl2-2 (EC 3.1.26.-); ATP-dependent helicase dcl2-2 (EC 3.6.4.-)]
**Q0CEI2**	*DCL2*	*Aspergillus terreus*	Ascomycota	242	25.30%	Dicer-like protein 2 [Includes: Endoribonuclease dcl2 (EC 3.1.26.-); ATP-dependent helicase dcl2 (EC 3.6.4.-)]
**Q6ATG6**	*RTL2*	*Oryza sativa (Rice)*	Streptophyta	241	28.40%	Ribonuclease 3-like protein 2 (EC 3.1.26.-) (Ribonuclease III-like protein 2) (RNase III-like protein 2)

**Q96C10**	*DHX58*	*Homo sapiens (Human)*	Chordata	239	25.70%	Probable ATP-dependent RNA helicase DHX58 (EC 3.6.4.13) (Probable ATP-dependent helicase LGP2) (Protein D11Lgp2 homolog) (RIG-I-like receptor 3) (RLR-3) (RIG-I-like receptor LGP2) (RLR)
**A7TSV4**	*MPH1*	*Vanderwaltozyma polyspora*	Ascomycota	229	21.60%	ATP-dependent DNA helicase MPH1 (EC 3.6.4.12) (FANCM-like protein 1)
**Q5A1A0**	*MPH1*	*Candida albicans (Yeast)*	Ascomycota	226	23.80%	ATP-dependent DNA helicase MPH1 (EC 3.6.4.12) (FANCM-like protein 1)
**Q9HE09**	*MFH2*	*Schizosaccharomyces pombe (Fission yeast)*	Ascomycota	225	22.10%	Putative ATP-dependent DNA helicase fml2 (EC 3.6.4.12) (FANCM-like protein 2)
**A6ZVS0**	*MPH1*	*Saccharomyces cerevisiae (Baker's yeast)*	Ascomycota	218	21.90%	ATP-dependent DNA helicase MPH1 (EC 3.6.4.12) (FANCM-like protein 1) (Mutator phenotype protein 1)
**Q7SCC1**	*DCL2*	*Neurospora crassa*	Ascomycota	215	28.80%	Dicer-like protein 2 [Includes: Endoribonuclease dcl-2 (EC 3.1.26.-); ATP-dependent helicase dcl-2 (EC 3.6.4.-)]
**A8EV88**	*RNC*	*Arcobacter butzleri*	Proteobacteria	214	27.20%	Ribonuclease 3 (EC 3.1.26.3) (Ribonuclease III) (RNase III)
**Q6CQX2**	*MPH1*	*Kluyveromyces lactis (Yeast) (Candida sphaerica)*	Ascomycota	208	24.30%	ATP-dependent DNA helicase MPH1 (EC 3.6.4.12) (FANCM-like protein 1)
**A6Q1H0**	*RNC*	*Nitratiruptor sp. (strain SB155-2)*	Proteobacteria	208	28.10%	Ribonuclease 3 (EC 3.1.26.3) (Ribonuclease III) (RNase III)
**P40562**	*MPH1*	*Saccharomyces cerevisiae (Baker's yeast)*	Ascomycota	208	21.70%	ATP-dependent DNA helicase MPH1 (EC 3.6.4.12) (FANCM-like protein 1) (Mutator phenotype protein 1)
**Q7M840**	*RNC*	*Wolinella succinogenes*	Proteobacteria	204	26.70%	Ribonuclease 3 (EC 3.1.26.3) (Ribonuclease III) (RNase III)
**A4IM67**	*RNC*	*Geobacillus thermodenitrificans*	Firmicutes	203	28.10%	Ribonuclease 3 (EC 3.1.26.3) (Ribonuclease III) (RNase III)
**Q819V8**	*RNC*	*Bacillus cereus*	Firmicutes	202	30.40%	Ribonuclease 3 (EC 3.1.26.3) (Ribonuclease III) (RNase III)
**B7HDX2**	*RNC*	*Bacillus cereus*	Firmicutes	202	30.40%	Ribonuclease 3 (EC 3.1.26.3) (Ribonuclease III) (RNase III)
**B7IUK8**	*RNC*	*Bacillus cereus*	Firmicutes	202	30.40%	Ribonuclease 3 (EC 3.1.26.3) (Ribonuclease III) (RNase III)
**A7GRH9**	*RNC*	*Bacillus cytotoxicus*	Firmicutes	202	30.40%	Ribonuclease 3 (EC 3.1.26.3) (Ribonuclease III) (RNase III)
**A6QCJ0**	*RNC*	*Sulfurovum sp. (strain NBC37-1)*	Proteobacteria	202	24.90%	Ribonuclease 3 (EC 3.1.26.3) (Ribonuclease III) (RNase III)
**Q9FKF0**	*RTL3*	*Arabidopsis thaliana (Mouse-ear cress)*	Streptophyta	200	26.30%	Ribonuclease 3-like protein 3 (EC 3.1.26.-) (Ribonuclease III-like protein 3) (RNase III-like protein 3) (Ribonuclease three-like protein 3)
**Q5L0Q3**	*RNC*	*Geobacillus kaustophilus*	Firmicutes	200	27.80%	Ribonuclease 3 (EC 3.1.26.3) (Ribonuclease III) (RNase III)
**B2G826**	*RNC*	*Lactobacillus reuteri*	Firmicutes	200	27.90%	Ribonuclease 3 (EC 3.1.26.3) (Ribonuclease III) (RNase III)
**A5VKP2**	*RNC*	*Lactobacillus reuteri*	Firmicutes	200	27.90%	Ribonuclease 3 (EC 3.1.26.3) (Ribonuclease III) (RNase III)
**A9VT87**	*RNC*	*Bacillus mycoides*	Firmicutes	199	29.80%	Ribonuclease 3 (EC 3.1.26.3) (Ribonuclease III) (RNase III)
**Q81WI8**	*RNC*	*Bacillus anthracis*	Firmicutes	198	30.40%	Ribonuclease 3 (EC 3.1.26.3) (Ribonuclease III) (RNase III)
**C3L778**	*RNC*	*Bacillus anthracis*	Firmicutes	198	30.40%	Ribonuclease 3 (EC 3.1.26.3) (Ribonuclease III) (RNase III)
**C3P5Q0**	*RNC*	*Bacillus anthracis*	Firmicutes	198	30.40%	Ribonuclease 3 (EC 3.1.26.3) (Ribonuclease III) (RNase III)
**B9IVD8**	*RNC*	*Bacillus cereus*	Firmicutes	198	30.40%	Ribonuclease 3 (EC 3.1.26.3) (Ribonuclease III) (RNase III)
**B7HLI2**	*RNC*	*Bacillus cereus*	Firmicutes	198	30.40%	Ribonuclease 3 (EC 3.1.26.3) (Ribonuclease III) (RNase III)
**C1EP73**	*RNC*	*Bacillus cereus*	Firmicutes	198	30.40%	Ribonuclease 3 (EC 3.1.26.3) (Ribonuclease III) (RNase III)

**Q732M1**	*RNC*	*Bacillus cereus*	Firmicutes	198	30.40%	Ribonuclease 3 (EC 3.1.26.3) (Ribonuclease III) (RNase III)
**B7JJT6**	*RNC*	*Bacillus cereus*	Firmicutes	198	30.40%	Ribonuclease 3 (EC 3.1.26.3) (Ribonuclease III) (RNase III)
**Q6HEW6**	*RNC*	*Bacillus thuringiensis*	Firmicutes	198	30.40%	Ribonuclease 3 (EC 3.1.26.3) (Ribonuclease III) (RNase III)
**Q8R5F7-2**	*IFIH1*	*Mus musculus (Mouse)*	Chordata	198	34.60%	Isoform 2 of Interferon-induced helicase C domain-containing protein 1
**Q8R5F7**	*IFIH1*	*Mus musculus (Mouse)*	Chordata	198	34.60%	Interferon-induced helicase C domain-containing protein 1 (EC 3.6.4.13) (Helicase with 2 CARD domains) (Helicard) (Interferon induced with helicase C domain protein 1) (Melanoma differentiation-associated protein 5) (MDA-5) (RIG-I-like receptor 2) (RLR-2)
**Q65JQ5**	*RNC*	*Bacillus licheniformis*	Firmicutes	197	28.60%	Ribonuclease 3 (EC 3.1.26.3) (Ribonuclease III) (RNase III)
**Q636H7**	*RNC*	*Bacillus cereus*	Firmicutes	196	30.40%	Ribonuclease 3 (EC 3.1.26.3) (Ribonuclease III) (RNase III)
**Q9KA05**	*RNC*	*Bacillus halodurans*	Firmicutes	196	27.80%	Ribonuclease 3 (EC 3.1.26.3) (Ribonuclease III) (RNase III)
**Q1WUA0**	*RNC*	*Lactobacillus salivarius*	Firmicutes	196	26.80%	Ribonuclease 3 (EC 3.1.26.3) (Ribonuclease III) (RNase III)
**Q9BYX4**	*IFIH1*	*Homo sapiens (Human)*	Chordata	195	34.00%	Interferon-induced helicase C domain-containing protein 1 (EC 3.6.4.13) (Clinically amyopathic dermatomyositis autoantigen 140 kDa) (CADM-140 autoantigen) (Helicase with 2 CARD domains) (Helicard) (Interferon-induced with helicase C domain protein 1) (Melanoma differentiation-associated protein 5) (MDA-5) (Murabutide down-regulated protein) (RIG-I-like receptor 2) (RLR-2) (RNA helicase-DEAD box protein 116)
**Q6FPQ3**	*MPH1*	*Candida glabrata (Yeast) (Torulopsis glabrata)*	Ascomycota	192	21.00%	ATP-dependent DNA helicase MPH1 (EC 3.6.4.12) (FANCM-like protein 1)
**Q88WK0**	*RNC*	*Lactobacillus plantarum*	Firmicutes	192	28.70%	Ribonuclease 3 (EC 3.1.26.3) (Ribonuclease III) (RNase III)
**B4U7T8**	*RNC*	*Hydrogenobaculum sp. (strain Y04AAS1)*	Aquificae	189	26.30%	Ribonuclease 3 (EC 3.1.26.3) (Ribonuclease III) (RNase III)
**Q38XR8**	*RNC*	*Lactobacillus sakei*	Firmicutes	189	28.60%	Ribonuclease 3 (EC 3.1.26.3) (Ribonuclease III) (RNase III)
**Q9UT23**	*MPH1*	*Schizosaccharomyces pombe (Fission yeast)*	Ascomycota	189	22.00%	ATP-dependent DNA helicase fml1 (EC 3.6.4.12) (FANCM-like protein 1)
**P51833**	*RNC*	*Bacillus subtilis*	Firmicutes	188	27.00%	Ribonuclease 3 (EC 3.1.26.3) (Ribonuclease III) (RNase III)
**Q8Y691**	*RNC*	*Listeria monocytogenes*	Firmicutes	187	25.00%	Ribonuclease 3 (EC 3.1.26.3) (Ribonuclease III) (RNase III)
**Q7VL75**	*RNC*	*Haemophilus ducreyi*	Proteobacteria	186	28.00%	Ribonuclease 3 (EC 3.1.26.3) (Ribonuclease III) (RNase III)
**B7GGE9**	*RNC*	*Anoxybacillus flavithermus*	Firmicutes	185	25.10%	Ribonuclease 3 (EC 3.1.26.3) (Ribonuclease III) (RNase III)
**C5D8T6**	*RNC*	*Geobacillus sp. (strain WCH70)*	Firmicutes	183	27.50%	Ribonuclease 3 (EC 3.1.26.3) (Ribonuclease III) (RNase III)
**Q92AK3**	*RNC*	*Listeria innocua*	Firmicutes	183	24.60%	Ribonuclease 3 (EC 3.1.26.3) (Ribonuclease III) (RNase III)
**Q71YL2**	*RNC*	*Listeria monocytogenes*	Firmicutes	183	24.60%	Ribonuclease 3 (EC 3.1.26.3) (Ribonuclease III) (RNase III)
**C1KWA4**	*RNC*	*Listeria monocytogenes*	Firmicutes	183	24.60%	Ribonuclease 3 (EC 3.1.26.3) (Ribonuclease III) (RNase III)
**B8DDU8**	*RNC*	*Listeria monocytogenes*	Firmicutes	182	24.60%	Ribonuclease 3 (EC 3.1.26.3) (Ribonuclease III) (RNase III)
**P74368**	*RNC*	*Synechocystis sp. PCC 6803*	Cyanobacteria	182	28.50%	Ribonuclease 3 1 (EC 3.1.26.3) (Ribonuclease III 1) (RNase III 1)

**Q6BRF0**	*MPH1*	*Debaryomyces hansenii (Yeast) (Torulaspora hansenii)*	Ascomycota	181	23.60%	ATP-dependent DNA helicase MPH1 (EC 3.6.4.12) (FANCM-like protein 1)
**A7Z4L3**	*RNC*	*Bacillus velezensis*	Firmicutes	180	26.60%	Ribonuclease 3 (EC 3.1.26.3) (Ribonuclease III) (RNase III)
**A8AWC2**	*RNC*	*Streptococcus gordonii*	Firmicutes	180	27.70%	Ribonuclease 3 (EC 3.1.26.3) (Ribonuclease III) (RNase III)
**B0USS1**	*RNC*	*Histophilus somni (Haemophilus somnus)*	Proteobacteria	179	27.30%	Ribonuclease 3 (EC 3.1.26.3) (Ribonuclease III) (RNase III)
**A0AJR0**	*RNC*	*Listeria welshimeri*	Firmicutes	179	24.20%	Ribonuclease 3 (EC 3.1.26.3) (Ribonuclease III) (RNase III)
**Q58900**	*Y1505*	*Methanocaldococcus jannaschii*	Euryarchaeota	179	22.90%	Putative ATP-dependent RNA helicase MJ1505 (EC 3.6.4.13)
**O67082**	*RNC*	*Aquifex aeolicus*	Aquificae	178	23.20%	Ribonuclease 3 (EC 3.1.26.3) (Ribonuclease III) (RNase III)
**Q6MLR5**	*RNC*	*Bdellovibrio bacteriovorus*	Proteobacteria	178	24.80%	Ribonuclease 3 (EC 3.1.26.3) (Ribonuclease III) (RNase III)
**B0BUA6**	*RNC*	*Actinobacillus pleuropneumoniae (Haemophilus pleuropneumoniae)*	Proteobacteria	177	26.80%	Ribonuclease 3 (EC 3.1.26.3) (Ribonuclease III) (RNase III)
**Q5WFM6**	*RNC*	*Bacillus clausii*	Firmicutes	177	25.70%	Ribonuclease 3 (EC 3.1.26.3) (Ribonuclease III) (RNase III)
**Q74IP8**	*RNC*	*Lactobacillus johnsonii*	Firmicutes	177	27.90%	Ribonuclease 3 (EC 3.1.26.3) (Ribonuclease III) (RNase III)
**A3MZQ9**	*RNC*	*Actinobacillus pleuropneumoniae (Haemophilus pleuropneumoniae)*	Proteobacteria	176	26.40%	Ribonuclease 3 (EC 3.1.26.3) (Ribonuclease III) (RNase III)
**A0Q7W6**	*RNC*	*Francisella tularensis*	Proteobacteria	176	26.70%	Ribonuclease 3 (EC 3.1.26.3) (Ribonuclease III) (RNase III)
**A8Z6F6**	*RNC*	*Campylobacter concisus*	Proteobacteria	175	24.10%	Ribonuclease 3 (EC 3.1.26.3) (Ribonuclease III) (RNase III)
**Q0BN07**	*RNC*	*Francisella tularensis*	Proteobacteria	175	26.70%	Ribonuclease 3 (EC 3.1.26.3) (Ribonuclease III) (RNase III)
**B2SFY4**	*RNC*	*Francisella tularensis*	Proteobacteria	175	26.70%	Ribonuclease 3 (EC 3.1.26.3) (Ribonuclease III) (RNase III)
**Q2A4N2**	*RNC*	*Francisella tularensis*	Proteobacteria	175	26.70%	Ribonuclease 3 (EC 3.1.26.3) (Ribonuclease III) (RNase III)
**A7NAR0**	*RNC*	*Francisella tularensis*	Proteobacteria	175	26.70%	Ribonuclease 3 (EC 3.1.26.3) (Ribonuclease III) (RNase III)
**A4J683**	*RNC*	*Desulfotomaculum reducens*	Firmicutes	174	28.10%	Ribonuclease 3 (EC 3.1.26.3) (Ribonuclease III) (RNase III)
**A4IWK9**	*RNC*	*Francisella tularensis*	Proteobacteria	174	26.70%	Ribonuclease 3 (EC 3.1.26.3) (Ribonuclease III) (RNase III)
**B2GD42**	*RNC*	*Lactobacillus fermentum*	Firmicutes	174	27.30%	Ribonuclease 3 (EC 3.1.26.3) (Ribonuclease III) (RNase III)
**Q8E0K7**	*RNC*	*Streptococcus agalactiae*	Firmicutes	174	27.60%	Ribonuclease 3 (EC 3.1.26.3) (Ribonuclease III) (RNase III)
**Q8E680**	*RNC*	*Streptococcus agalactiae*	Firmicutes	174	27.60%	Ribonuclease 3 (EC 3.1.26.3) (Ribonuclease III) (RNase III)
**Q3K1Y2**	*RNC*	*Streptococcus agalactiae*	Firmicutes	174	27.60%	Ribonuclease 3 (EC 3.1.26.3) (Ribonuclease III) (RNase III)
**Q03FX0**	*RNC*	*Pediococcus pentosaceus*	Firmicutes	173	26.80%	Ribonuclease 3 (EC 3.1.26.3) (Ribonuclease III) (RNase III)
**Q6Q899-3**	*DDX58*	*Mus musculus (Mouse)*	Chordata	172	32.10%	Isoform 3 of Probable ATP-dependent RNA helicase DDX58
**Q99J87**	*DHX58*	*Mus musculus (Mouse)*	Chordata	172	36.50%	Probable ATP-dependent RNA helicase DHX58 (EC 3.6.4.13) (Probable ATP-dependent helicase LGP2) (Protein D11Lgp2) (RIG-I-like receptor 3) (RLR-3) (RIG-I-like receptor Lgp2) (RLR)
**Q9XCX9**	*RNC*	*Pseudomonas aeruginosa*	Proteobacteria	172	27.40%	Ribonuclease 3 (EC 3.1.26.3) (Ribonuclease III) (RNase III)
**Q02HS2**	*RNC*	*Pseudomonas aeruginosa*	Proteobacteria	172	27.40%	Ribonuclease 3 (EC 3.1.26.3) (Ribonuclease III) (RNase III)

**B7UYX2**	*RNC*	*Pseudomonas aeruginosa*	Proteobacteria	172	27.40%	Ribonuclease 3 (EC 3.1.26.3) (Ribonuclease III) (RNase III)
**Q9ZBQ7**	*RNC*	*Streptomyces coelicolor*	Actinobacteria	172	27.30%	Ribonuclease 3 (EC 3.1.26.3) (Antibiotic biosynthesis protein B) (AbsB) (Ribonuclease III) (RNase III)
**Q3AC58**	*RNC*	*Carboxydothermus hydrogenoformans*	Firmicutes	170	24.90%	Ribonuclease 3 (EC 3.1.26.3) (Ribonuclease III) (RNase III)
**Q2SL32**	*RNC*	*Hahella chejuensis*	Proteobacteria	170	24.10%	Ribonuclease 3 (EC 3.1.26.3) (Ribonuclease III) (RNase III)
**Q4A589**	*RNC*	*Mycoplasma synoviae*	Tenericutes	170	25.10%	Ribonuclease 3 (EC 3.1.26.3) (Ribonuclease III) (RNase III)
**A6VAK6**	*RNC*	*Pseudomonas aeruginosa*	Proteobacteria	170	27.70%	Ribonuclease 3 (EC 3.1.26.3) (Ribonuclease III) (RNase III)
**A0RRM7**	*RNC*	*Campylobacter fetus*	Proteobacteria	169	23.90%	Ribonuclease 3 (EC 3.1.26.3) (Ribonuclease III) (RNase III)
**Q82JT9**	*RNC*	*Streptomyces avermitilis*	Actinobacteria	169	26.70%	Ribonuclease 3 (EC 3.1.26.3) (Ribonuclease III) (RNase III)
**Q5NER3**	*RNC*	*Francisella tularensis*	Proteobacteria	168	26.30%	Ribonuclease 3 (EC 3.1.26.3) (Ribonuclease III) (RNase III)
**Q14G66**	*RNC*	*Francisella tularensis*	Proteobacteria	168	26.30%	Ribonuclease 3 (EC 3.1.26.3) (Ribonuclease III) (RNase III)
**B8F3C7**	*RNC*	*Haemophilus parasuis*	Proteobacteria	168	26.40%	Ribonuclease 3 (EC 3.1.26.3) (Ribonuclease III) (RNase III)
**Q5ZUD5**	*RNC*	*Legionella pneumophila*	Proteobacteria	168	27.10%	Ribonuclease 3 (EC 3.1.26.3) (Ribonuclease III) (RNase III)
**B0KV27**	*RNC*	*Pseudomonas putida (Arthrobacter siderocapsulatus)*	Proteobacteria	168	28.00%	Ribonuclease 3 (EC 3.1.26.3) (Ribonuclease III) (RNase III)
**C0MCR4**	*RNC*	*Streptococcus equi*	Firmicutes	168	26.10%	Ribonuclease 3 (EC 3.1.26.3) (Ribonuclease III) (RNase III)
**Q8R9W3**	*RNC*	*Caldanaerobacter subterraneus*	Firmicutes	167	24.90%	Ribonuclease 3 (EC 3.1.26.3) (Ribonuclease III) (RNase III)
**Q6F1N5**	*RNC*	*Mesoplasma florum (Acholeplasma florum)*	Tenericutes	167	27.20%	Ribonuclease 3 (EC 3.1.26.3) (Ribonuclease III) (RNase III)
**C0M8Y3**	*RNC*	*Streptococcus equi*	Firmicutes	167	26.10%	Ribonuclease 3 (EC 3.1.26.3) (Ribonuclease III) (RNase III)
**B9JUN7**	*RNC*	*Agrobacterium vitis (Rhizobium vitis)*	Proteobacteria	166	26.30%	Ribonuclease 3 (EC 3.1.26.3) (Ribonuclease III) (RNase III)
**Q5HSF8**	*RNC*	*Campylobacter jejuni*	Proteobacteria	166	28.70%	Ribonuclease 3 (EC 3.1.26.3) (Ribonuclease III) (RNase III)
**Q9PM40**	*RNC*	*Campylobacter jejuni*	Proteobacteria	166	28.70%	Ribonuclease 3 (EC 3.1.26.3) (Ribonuclease III) (RNase III)
**C4K3Z3**	*RNC*	*Candidatus Hamiltonella defensa*	Proteobacteria	166	23.30%	Ribonuclease 3 (EC 3.1.26.3) (Ribonuclease III) (RNase III)
**B0TXH8**	*RNC*	*Francisella philomiragia*	Proteobacteria	166	29.30%	Ribonuclease 3 (EC 3.1.26.3) (Ribonuclease III) (RNase III)
**Q39T82**	*RNC*	*Geobacter metallireducens*	Proteobacteria	166	26.60%	Ribonuclease 3 (EC 3.1.26.3) (Ribonuclease III) (RNase III)
**A8FNU7**	*RNC*	*Campylobacter jejuni*	Proteobacteria	165	28.70%	Ribonuclease 3 (EC 3.1.26.3) (Ribonuclease III) (RNase III)
**A7H5Y2**	*RNC*	*Campylobacter jejuni*	Proteobacteria	165	28.70%	Ribonuclease 3 (EC 3.1.26.3) (Ribonuclease III) (RNase III)
**Q7VIA9**	*RNC*	*Helicobacter hepaticus*	Proteobacteria	165	24.30%	Ribonuclease 3 (EC 3.1.26.3) (Ribonuclease III) (RNase III)
**Q5FJJ7**	*RNC*	*Lactobacillus acidophilus*	Firmicutes	165	25.00%	Ribonuclease 3 (EC 3.1.26.3) (Ribonuclease III) (RNase III)
**Q1I5V8**	*RNC*	*Pseudomonas entomophila*	Proteobacteria	165	27.80%	Ribonuclease 3 (EC 3.1.26.3) (Ribonuclease III) (RNase III)
**Q88MY5**	*RNC*	*Pseudomonas putida (Arthrobacter siderocapsulatus)*	Proteobacteria	164	28.50%	Ribonuclease 3 (EC 3.1.26.3) (Ribonuclease III) (RNase III)
**A5W8F2**	*RNC*	*Pseudomonas putida (Arthrobacter siderocapsulatus)*	Proteobacteria	164	28.50%	Ribonuclease 3 (EC 3.1.26.3) (Ribonuclease III) (RNase III)

**A8F397**	*RNC*	*Pseudothermotoga lettingae*	Thermotogae	164	24.10%	Ribonuclease 3 (EC 3.1.26.3) (Ribonuclease III) (RNase III)
**Q1RHA2**	*RNC*	*Rickettsia bellii*	Proteobacteria	164	24.80%	Ribonuclease 3 (EC 3.1.26.3) (Ribonuclease III) (RNase III)
**A8GYE2**	*RNC*	*Rickettsia bellii*	Proteobacteria	164	24.80%	Ribonuclease 3 (EC 3.1.26.3) (Ribonuclease III) (RNase III)
**C1CEK4**	*RNC*	*Streptococcus pneumoniae*	Firmicutes	164	26.70%	Ribonuclease 3 (EC 3.1.26.3) (Ribonuclease III) (RNase III)
**Q8DPJ8**	*RNC*	*Streptococcus pneumoniae*	Firmicutes	164	26.70%	Ribonuclease 3 (EC 3.1.26.3) (Ribonuclease III) (RNase III)
**B2IPN5**	*RNC*	*Streptococcus pneumoniae*	Firmicutes	164	26.70%	Ribonuclease 3 (EC 3.1.26.3) (Ribonuclease III) (RNase III)
**Q97QG6**	*RNC*	*Streptococcus pneumoniae*	Firmicutes	164	26.70%	Ribonuclease 3 (EC 3.1.26.3) (Ribonuclease III) (RNase III)
**C1C7M6**	*RNC*	*Streptococcus pneumoniae*	Firmicutes	164	26.70%	Ribonuclease 3 (EC 3.1.26.3) (Ribonuclease III) (RNase III)
**B5E4Y2**	*RNC*	*Streptococcus pneumoniae*	Firmicutes	164	26.70%	Ribonuclease 3 (EC 3.1.26.3) (Ribonuclease III) (RNase III)
**Q04K72**	*RNC*	*Streptococcus pneumoniae*	Firmicutes	164	26.70%	Ribonuclease 3 (EC 3.1.26.3) (Ribonuclease III) (RNase III)
**Q5WVI4**	*RNC*	*Legionella pneumophila*	Proteobacteria	163	26.70%	Ribonuclease 3 (EC 3.1.26.3) (Ribonuclease III) (RNase III)
**C1CR56**	*RNC*	*Streptococcus pneumoniae*	Firmicutes	163	26.70%	Ribonuclease 3 (EC 3.1.26.3) (Ribonuclease III) (RNase III)
**B8ZJR8**	*RNC*	*Streptococcus pneumoniae*	Firmicutes	163	26.70%	Ribonuclease 3 (EC 3.1.26.3) (Ribonuclease III) (RNase III)
**B1IC43**	*RNC*	*Streptococcus pneumoniae*	Firmicutes	163	26.70%	Ribonuclease 3 (EC 3.1.26.3) (Ribonuclease III) (RNase III)
**A5ID21**	*RNC*	*Legionella pneumophila*	Proteobacteria	162	26.70%	Ribonuclease 3 (EC 3.1.26.3) (Ribonuclease III) (RNase III)
**Q5X446**	*RNC*	*Legionella pneumophila*	Proteobacteria	162	26.70%	Ribonuclease 3 (EC 3.1.26.3) (Ribonuclease III) (RNase III)
**Q3J8D5**	*RNC*	*Nitrosococcus oceani*	Proteobacteria	162	27.70%	Ribonuclease 3 (EC 3.1.26.3) (Ribonuclease III) (RNase III)
**Q8ER05**	*RNC*	*Oceanobacillus iheyensis*	Firmicutes	162	23.90%	Ribonuclease 3 (EC 3.1.26.3) (Ribonuclease III) (RNase III)
**Q4KHT1**	*RNC*	*Pseudomonas protegens*	Proteobacteria	161	27.80%	Ribonuclease 3 (EC 3.1.26.3) (Ribonuclease III) (RNase III)
**C1CKY7**	*RNC*	*Streptococcus pneumoniae*	Firmicutes	161	26.30%	Ribonuclease 3 (EC 3.1.26.3) (Ribonuclease III) (RNase III)
**B0BBS8**	*RNC*	*Chlamydia trachomatis*	Chlamydiae	160	26.10%	Ribonuclease 3 (EC 3.1.26.3) (Ribonuclease III) (RNase III)
**B1J4E0**	*RNC*	*Pseudomonas putida (Arthrobacter siderocapsulatus)*	Proteobacteria	160	28.10%	Ribonuclease 3 (EC 3.1.26.3) (Ribonuclease III) (RNase III)
**Q68XY5**	*RNC*	*Rickettsia typhi*	Proteobacteria	160	26.70%	Ribonuclease 3 (EC 3.1.26.3) (Ribonuclease III) (RNase III)
**Q7WD32**	*RNC*	*Bordetella bronchiseptica (Alcaligenes bronchisepticus)*	Proteobacteria	159	24.50%	Ribonuclease 3 (EC 3.1.26.3) (Ribonuclease III) (RNase III)
**Q7W5J6**	*RNC*	*Bordetella parapertussis*	Proteobacteria	159	24.50%	Ribonuclease 3 (EC 3.1.26.3) (Ribonuclease III) (RNase III)
**Q7VW39**	*RNC*	*Bordetella pertussis*	Proteobacteria	159	24.50%	Ribonuclease 3 (EC 3.1.26.3) (Ribonuclease III) (RNase III)
**B6J4J9**	*RNC*	*Coxiella burnetii*	Proteobacteria	159	27.70%	Ribonuclease 3 (EC 3.1.26.3) (Ribonuclease III) (RNase III)
**Q8IYD8-2**	*FANCM*	*Homo sapiens (Human)*	Chordata	159	31.30%	Isoform 2 of Fanconi anemia group M protein
**Q8IYD8**	*FANCM*	*Homo sapiens (Human)*	Chordata	159	31.30%	Fanconi anemia group M protein (Protein FACM) (EC 3.6.4.13) (ATP-dependent RNA helicase FANCM) (Fanconi anemia-associated polypeptide of 250 kDa) (FAAP250) (Protein Hef ortholog)
**A8EXI3**	*RNC*	*Rickettsia canadensis*	Proteobacteria	159	25.20%	Ribonuclease 3 (EC 3.1.26.3) (Ribonuclease III) (RNase III)

**B0B7L3**	*RNC*	*Chlamydia trachomatis*	Chlamydiae	158	26.10%	Ribonuclease 3 (EC 3.1.26.3) (Ribonuclease III) (RNase III)
**Q9NRR4-2**	*RNC*	*Homo sapiens (Human)*	Chordata	158	26.20%	Isoform 2 of Ribonuclease 3
**Q9NRR4-4**	*RNC*	*Homo sapiens (Human)*	Chordata	158	26.20%	Isoform 4 of Ribonuclease 3
**Q9NRR4**	*RNC*	*Homo sapiens (Human)*	Chordata	158	26.20%	Ribonuclease 3 (EC 3.1.26.3) (Protein Drosha) (Ribonuclease III) (RNase III) (p241)
**Q5HZJ0**	*RNC*	*Mus musculus (Mouse)*	Chordata	158	26.20%	Ribonuclease 3 (EC 3.1.26.3) (Protein Drosha) (Ribonuclease III) (RNase III)

Table S3. Detailed sequence information of TRBP members used for constructing a phylogenetic tree.

Entry	Gene names	Taxonomic lineage (SPECIES)	Taxonomic lineage (PHYLUM)	Score	Identity	Protein names
**Q15633**	*TARBP2*	*Homo sapiens (Human)*	Chordata	1,889	100.00%	RISC-loading complex subunit TARBP2 (TAR RNA-binding protein 2) (Trans-activation-responsive RNA-binding protein)
**Q0IIG6**	*TARBP2*	*Bos taurus (Bovine)*	Chordata	1,847	97.50%	RISC-loading complex subunit TARBP2
**Q15633-2**	*TARBP2*	*Homo sapiens (Human)*	Chordata	1,779	100.00%	Isoform 2 of RISC-loading complex subunit TARBP2 (Isoform TRBP1 of RISC-loading complex subunit TARBP2)
**P97473**	*TARBP2*	*Mus musculus (Mouse)*	Chordata	1,775	94.50%	RISC-loading complex subunit TARBP2 (Protamine-1 RNA-binding protein) (PRM-1 RNA-binding protein) (TAR RNA-binding protein 2)
**Q3SWU0**	*TARBP2*	*Rattus norvegicus (Rat)*	Chordata	1,756	92.90%	RISC-loading complex subunit TARBP2
**Q6GPZ1**	*TARBP2*	*Xenopus laevis (African clawed frog)*	Chordata	1,235	70.10%	RISC-loading complex subunit tarbp2
**Q5BJ52**	*TARBP2*	*Xenopus tropicalis (Western clawed frog) (Silurana tropicalis)*	Chordata	1,224	69.60%	RISC-loading complex subunit tarbp2
**Q7SXR1**	*TARBP2*	*Danio rerio (Zebrafish) (Brachydanio rerio)*	Chordata	1,155	64.40%	RISC-loading complex subunit tarbp2
**Q4SS66**	*TARBP2*	*Tetraodon nigroviridis (Spotted green pufferfish) (Chelonodon nigroviridis)*	Chordata	1,132	64.30%	RISC-loading complex subunit tarbp2
**Q7ZYA5**	*PRKRA*	*Xenopus laevis (African clawed frog)*	Chordata	715	45.80%	Interferon-inducible double-stranded RNA-dependent protein kinase activator A homolog A
**Q91836**	*PRKRA*	*Xenopus laevis (African clawed frog)*	Chordata	708	45.60%	Interferon-inducible double-stranded RNA-dependent protein kinase activator A homolog B (Double-stranded RNA-binding protein A) (XlRBPA)
**O75569-2**	*PRKRA*	*Homo sapiens (Human)*	Chordata	677	42.30%	Isoform 2 of Interferon-inducible double-stranded RNA-dependent protein kinase activator A
**Q2HJ92**	*PRKRA*	*Bos taurus (Bovine)*	Chordata	674	42.10%	Interferon-inducible double-stranded RNA-dependent protein kinase activator A (Protein activator of the interferon-induced protein kinase) (Protein kinase, interferon-inducible double-stranded RNA-dependent activator)
**Q4V8C7**	*PRKRA*	*Rattus norvegicus (Rat)*	Chordata	673	43.80%	Interferon-inducible double-stranded RNA-dependent protein kinase activator A (Protein activator of the interferon-induced protein kinase) (Protein kinase, interferon-inducible double-stranded RNA-dependent activator)
**Q9WTX2**	*PRKRA*	*Mus musculus (Mouse)*	Chordata	668	43.50%	Interferon-inducible double-stranded RNA-dependent protein kinase activator A (PKR-associated protein X) (PKR-associating protein X) (RAX) (Protein activator of the interferon-induced protein kinase) (Protein kinase, interferon-inducible double-stranded RNA-dependent activator)
**O75569**	*PRKRA*	*Homo sapiens (Human)*	Chordata	665	41.90%	Interferon-inducible double-stranded RNA-dependent protein kinase activator A (PKR-associated protein X) (PKR-associating protein X) (Protein activator of the interferon-induced protein kinase) (Protein kinase, interferon-inducible double-stranded

						RNA-dependent activator)
**O75569-3**	*PRKRA*	*Homo sapiens (Human)*	Chordata	658	42.60%	Isoform 3 of Interferon-inducible double-stranded RNA-dependent protein kinase activator A
**B0V3F8**	*PRKRA*	*Danio rerio (Zebrafish) (Brachydanio rerio)*	Chordata	553	39.60%	Interferon-inducible double-stranded RNA-dependent protein kinase activator A homolog
**Q5M7M7**	*STAU2*	*Xenopus tropicalis (Western clawed frog) (Silurana tropicalis)*	Chordata	210	29.70%	Double-stranded RNA-binding protein Staufen homolog 2
**Q7ZW47**	*STAU2*	*Danio rerio (Zebrafish) (Brachydanio rerio)*	Chordata	201	30.70%	Double-stranded RNA-binding protein Staufen homolog 2
**Q9NUL3-4**	*STAU2*	*Homo sapiens (Human)*	Chordata	194	28.80%	Isoform 4 of Double-stranded RNA-binding protein Staufen homolog 2
**Q9NUL3-6**	*STAU2*	*Homo sapiens (Human)*	Chordata	194	28.80%	Isoform 6 of Double-stranded RNA-binding protein Staufen homolog 2
**Q9NUL3-8**	*STAU2*	*Homo sapiens (Human)*	Chordata	194	28.80%	Isoform 8 of Double-stranded RNA-binding protein Staufen homolog 2
**Q9NUL3-3**	*STAU2*	*Homo sapiens (Human)*	Chordata	194	28.80%	Isoform 3 of Double-stranded RNA-binding protein Staufen homolog 2
**Q9NUL3-7**	*STAU2*	*Homo sapiens (Human)*	Chordata	194	28.80%	Isoform 7 of Double-stranded RNA-binding protein Staufen homolog 2
**Q9NUL3-2**	*STAU2*	*Homo sapiens (Human)*	Chordata	194	28.80%	Isoform 2 of Double-stranded RNA-binding protein Staufen homolog 2 (Isoform Short of Double-stranded RNA-binding protein Staufen homolog 2)
**Q9NUL3**	*STAU2*	*Homo sapiens (Human)*	Chordata	194	28.80%	Double-stranded RNA-binding protein Staufen homolog 2
**Q68SB1-3**	*STAU2*	*Rattus norvegicus (Rat)*	Chordata	193	28.30%	Isoform 3 of Double-stranded RNA-binding protein Staufen homolog 2 (Isoform SS of Double-stranded RNA-binding protein Staufen homolog 2)
**Q68SB1-4**	*STAU2*	*Rattus norvegicus (Rat)*	Chordata	193	28.30%	Isoform 4 of Double-stranded RNA-binding protein Staufen homolog 2 (Isoform LS of Double-stranded RNA-binding protein Staufen homolog 2) (Isoform B of Double-stranded RNA-binding protein Staufen homolog 2)
**Q68SB1-2**	*STAU2*	*Rattus norvegicus (Rat)*	Chordata	193	28.30%	Isoform 2 of Double-stranded RNA-binding protein Staufen homolog 2 (Isoform SL of Double-stranded RNA-binding protein Staufen homolog 2)
**Q68SB1**	*STAU2*	*Rattus norvegicus (Rat)*	Chordata	193	28.30%	Double-stranded RNA-binding protein Staufen homolog 2 (r-staufen protein)
**O95793-2**	*STAU1*	*Homo sapiens (Human)*	Chordata	192	29.30%	Isoform Short of Double-stranded RNA-binding protein Staufen homolog 1
**O95793**	*STAU1*	*Homo sapiens (Human)*	Chordata	192	29.30%	Double-stranded RNA-binding protein Staufen homolog 1
**Q8CJ67-3**	*STAU2*	*Mus musculus (Mouse)*	Chordata	188	28.40%	Isoform 3 of Double-stranded RNA-binding protein Staufen homolog 2
**O95793-3**	*STAU1*	*Homo sapiens (Human)*	Chordata	188	29.20%	Isoform 3 of Double-stranded RNA-binding protein Staufen homolog 1
**Q8CJ67-2**	*STAU2*	*Mus musculus (Mouse)*	Chordata	188	28.40%	Isoform 2 of Double-stranded RNA-binding protein Staufen homolog 2 (Isoform Short of Double-stranded RNA-binding protein Staufen homolog 2)
**Q8CJ67**	*STAU2*	*Mus musculus (Mouse)*	Chordata	188	28.40%	Double-stranded RNA-binding protein Staufen homolog 2
**Q3L1C9**	*STAUH*	*Aplysia californica (California sea hare)*	Mollusca	184	28.90%	Double-stranded RNA-binding protein Staufen homolog
**P78563-6**	*RED1*	*Homo sapiens (Human)*	Chordata	177	29.50%	Isoform 6 of Double-stranded RNA-specific editase 1 (Isoform ADAR2d of Double-stranded RNA-specific editase 1)

**P78563-2**	*RED1*	*Homo sapiens (Human)*	Chordata	177	29.50%	Isoform 2 of Double-stranded RNA-specific editase 1 (Isoform ADAR2a of Double-stranded RNA-specific editase 1) (Isoform DRADA2A of Double-stranded RNA-specific editase 1) (Isoform RED1-S of Double-stranded RNA-specific editase 1)
**P78563-3**	*RED1*	*Homo sapiens (Human)*	Chordata	177	29.50%	Isoform 3 of Double-stranded RNA-specific editase 1 (Isoform DRADA2C of Double-stranded RNA-specific editase 1)
**P78563-4**	*RED1*	*Homo sapiens (Human)*	Chordata	177	29.50%	Isoform 4 of Double-stranded RNA-specific editase 1
**P78563**	*RED1*	*Homo sapiens (Human)*	Chordata	177	29.50%	Double-stranded RNA-specific editase 1 (EC 3.5.4.37) (RNA-editing deaminase 1) (RNA-editing enzyme 1) (dsRNA adenosine deaminase)
**P78563-5**	*RED1*	*Homo sapiens (Human)*	Chordata	177	29.50%	Isoform 5 of Double-stranded RNA-specific editase 1 (Isoform ADAR2R of Double-stranded RNA-specific editase 1)
**Q91ZS8-5**	*RED1*	*Mus musculus (Mouse)*	Chordata	176	31.00%	Isoform 5 of Double-stranded RNA-specific editase 1
**Q91ZS8-4**	*RED1*	*Mus musculus (Mouse)*	Chordata	176	31.00%	Isoform 4 of Double-stranded RNA-specific editase 1
**Q91ZS8-3**	*RED1*	*Mus musculus (Mouse)*	Chordata	176	31.00%	Isoform 3 of Double-stranded RNA-specific editase 1
**Q91ZS8-2**	*RED1*	*Mus musculus (Mouse)*	Chordata	176	31.00%	Isoform 2 of Double-stranded RNA-specific editase 1
**P51400**	*RED1*	*Rattus norvegicus (Rat)*	Chordata	176	31.00%	Double-stranded RNA-specific editase 1 (EC 3.5.4.37) (RNA-editing deaminase 1) (RNA-editing enzyme 1) (dsRNA adenosine deaminase)
**Q91ZS8**	*RED1*	*Mus musculus (Mouse)*	Chordata	176	31.00%	Double-stranded RNA-specific editase 1 (EC 3.5.4.37) (RNA-editing deaminase 1) (RNA-editing enzyme 1) (dsRNA adenosine deaminase)
**Q91ZS8-6**	*RED1*	*Mus musculus (Mouse)*	Chordata	176	31.00%	Isoform 6 of Double-stranded RNA-specific editase 1
**D2GVP7**	*STAU1*	*Ailuropoda melanoleuca (Giant panda)*	Chordata	174	29.40%	Double-stranded RNA-binding protein Staufen homolog 1
**P25159-2**	*STAU*	*Drosophila melanogaster (Fruit fly)*	Arthropoda	173	28.50%	Isoform B of Maternal effect protein staufen
**P25159**	*STAU*	*Drosophila melanogaster (Fruit fly)*	Arthropoda	173	28.50%	Maternal effect protein staufen
**Q9Z108**	*STAU1*	*Mus musculus (Mouse)*	Chordata	173	28.90%	Double-stranded RNA-binding protein Staufen homolog 1
**P55265-5**	*DSRAD*	*Homo sapiens (Human)*	Chordata	164	29.60%	Isoform 5 of Double-stranded RNA-specific adenosine deaminase (Isoform ADAR1S of Double-stranded RNA-specific adenosine deaminase) (Isoform p110 of Double-stranded RNA-specific adenosine deaminase)
**P55265-2**	*DSRAD*	*Homo sapiens (Human)*	Chordata	164	29.60%	Isoform 2 of Double-stranded RNA-specific adenosine deaminase (Isoform ADAR-b of Double-stranded RNA-specific adenosine deaminase)
**P55265**	*DSRAD*	*Homo sapiens (Human)*	Chordata	164	29.60%	Double-stranded RNA-specific adenosine deaminase (DRADA) (EC 3.5.4.37) (136 kDa double-stranded RNA-binding protein) (p136) (Interferon-inducible protein 4) (IFI-4) (K88DSRBP)
**P55265-4**	*DSRAD*	*Homo sapiens (Human)*	Chordata	164	29.60%	Isoform 4 of Double-stranded RNA-specific adenosine deaminase
**P55266**	*DSRAD*	*Rattus norvegicus (Rat)*	Chordata	153	30.10%	Double-stranded RNA-specific adenosine deaminase (DRADA) (EC 3.5.4.37)
**Q99MU3-3**	*DSRAD*	*Mus musculus (Mouse)*	Chordata	151	29.50%	Isoform 3 of Double-stranded RNA-specific adenosine deaminase (Isoform ADAR1Sa of Double-stranded RNA-specific adenosine deaminase)

**Q99MU3-4**	*DSRAD*	*Mus musculus (Mouse)*	Chordata	151	29.50%	Isoform 4 of Double-stranded RNA-specific adenosine deaminase (Isoform ADAR1Sb of Double-stranded RNA-specific adenosine deaminase) (Isoform p80 of Double-stranded RNA-specific adenosine deaminase)
**Q99MU3-5**	*DSRAD*	*Mus musculus (Mouse)*	Chordata	151	29.50%	Isoform 5 of Double-stranded RNA-specific adenosine deaminase (Isoform p110 of Double-stranded RNA-specific adenosine deaminase)
**Q99MU3-2**	*DSRAD*	*Mus musculus (Mouse)*	Chordata	151	29.50%	Isoform 2 of Double-stranded RNA-specific adenosine deaminase (Isoform ADAR1La of Double-stranded RNA-specific adenosine deaminase)
**Q99MU3**	*DSRAD*	*Mus musculus (Mouse)*	Chordata	151	29.50%	Double-stranded RNA-specific adenosine deaminase (DRADA) (EC 3.5.4.37) (RNA adenosine deaminase 1)
**Q91WM1-2**	*STRBP*	*Mus musculus (Mouse)*	Chordata	148	27.20%	Isoform 2 of Spermatid perinuclear RNA-binding protein
**Q91WM1**	*STRBP*	*Mus musculus (Mouse)*	Chordata	148	27.20%	Spermatid perinuclear RNA-binding protein
**Q91550-2**	*ILF3A*	*Xenopus laevis (African clawed frog)*	Chordata	147	30.70%	Isoform 2 of Interleukin enhancer-binding factor 3-A (Isoform CBTF98 of Interleukin enhancer-binding factor 3-A)
**Q91550**	*ILF3A*	*Xenopus laevis (African clawed frog)*	Chordata	147	30.70%	Interleukin enhancer-binding factor 3-A (CCAAT box transcription factor subunit) (Double-stranded RNA-binding protein 4F.1) (DsRNA-binding protein 4F.1)
**Q96SI9-2**	*STRBP*	*Homo sapiens (Human)*	Chordata	145	26.70%	Isoform 2 of Spermatid perinuclear RNA-binding protein
**Q5R6Y5**	*STRBP*	*Pongo abelii (Sumatran orangutan) (Pongo pygmaeus abelii)*	Chordata	145	26.70%	Spermatid perinuclear RNA-binding protein
**Q96SI9**	*STRBP*	*Homo sapiens (Human)*	Chordata	145	26.70%	Spermatid perinuclear RNA-binding protein
**Q08E27**	*STRBP*	*Bos taurus (Bovine)*	Chordata	143	26.70%	Spermatid perinuclear RNA-binding protein
**P55265-3**	*DSRAD*	*Homo sapiens (Human)*	Chordata	143	28.50%	Isoform 3 of Double-stranded RNA-specific adenosine deaminase (Isoform ADAR-c of Double-stranded RNA-specific adenosine deaminase)
**Q5ZIL4**	*STRBP*	*Gallus gallus (Chicken)*	Chordata	140	27.10%	Spermatid perinuclear RNA-binding protein
**Q12906-5**	*ILF3*	*Homo sapiens (Human)*	Chordata	140	30.80%	Isoform 5 of Interleukin enhancer-binding factor 3 (Isoform DRBP76 Delta of Interleukin enhancer-binding factor 3) (Isoform Gamma of Interleukin enhancer-binding factor 3) (Isoform ILF3-C of Interleukin enhancer-binding factor 3)
**Q12906-2**	*ILF3*	*Homo sapiens (Human)*	Chordata	140	30.80%	Isoform 2 of Interleukin enhancer-binding factor 3 (Isoform NFAR-1 of Interleukin enhancer-binding factor 3) (Isoform DRBP76 of Interleukin enhancer-binding factor 3)
**Q12906-3**	*ILF3*	*Homo sapiens (Human)*	Chordata	140	30.80%	Isoform 3 of Interleukin enhancer-binding factor 3
**Q12906**	*ILF3*	*Homo sapiens (Human)*	Chordata	140	30.80%	Interleukin enhancer-binding factor 3 (Double-stranded RNA-binding protein 76) (DRBP76) (M-phase phosphoprotein 4) (MPP4) (Nuclear factor associated with dsRNA) (NFAR) (Nuclear factor of activated T-cells 90 kDa) (NF-AT-90) (Translational control protein 80) (TCP80)
**Q12906-4**	*ILF3*	*Homo sapiens (Human)*	Chordata	139	30.20%	Isoform 4 of Interleukin enhancer-binding factor 3 (Isoform DRBP76 Alpha of Interleukin enhancer-binding factor 3) (Isoform ILF3-A of Interleukin enhancer-binding factor 3)
**Q12906-6**	*ILF3*	*Homo sapiens (Human)*	Chordata	139	30.20%	Isoform 6 of Interleukin enhancer-binding factor 3

**Q12906-7**	*ILF3*	*Homo sapiens (Human)*	Chordata	139	30.20%	Isoform 7 of Interleukin enhancer-binding factor 3
**Q5P9U8**	*RNC*	*Anaplasma marginale*	Proteobacteria	137	34.80%	Ribonuclease 3 (EC 3.1.26.3) (Ribonuclease III) (RNase III)
**B9KGT5**	*RNC*	*Anaplasma marginale*	Proteobacteria	137	34.80%	Ribonuclease 3 (EC 3.1.26.3) (Ribonuclease III) (RNase III)
**Q6DCD0**	*STRBP*	*Xenopus laevis (African clawed frog)*	Chordata	135	30.20%	Spermatid perinuclear RNA-binding protein
**P97616**	*RED2*	*Rattus norvegicus (Rat)*	Chordata	134	30.60%	Double-stranded RNA-specific editase B2 (EC 3.5.-.-) (RNA-dependent adenosine deaminase 3) (RNA-editing deaminase 2) (RNA-editing enzyme 2) (dsRNA adenosine deaminase B2)
**Q7VL75**	*RNC*	*Haemophilus ducreyi*	Proteobacteria	134	37.20%	Ribonuclease 3 (EC 3.1.26.3) (Ribonuclease III) (RNase III)
**Q6DD04-2**	*ILF3B*	*Xenopus laevis (African clawed frog)*	Chordata	133	30.90%	Isoform 2 of Interleukin enhancer-binding factor 3-B
**Q6GL57**	*ILF3*	*Xenopus tropicalis (Western clawed frog) (Silurana tropicalis)*	Chordata	133	28.60%	Interleukin enhancer-binding factor 3
**Q9JI20**	*RED2*	*Mus musculus (Mouse)*	Chordata	133	30.60%	Double-stranded RNA-specific editase B2 (EC 3.5.-.-) (RNA-dependent adenosine deaminase 3) (RNA-editing deaminase 2) (RNA-editing enzyme 2) (dsRNA adenosine deaminase B2)
**Q6DD04**	*ILF3B*	*Xenopus laevis (African clawed frog)*	Chordata	133	30.90%	Interleukin enhancer-binding factor 3-B (Double-stranded RNA-binding protein 4F.2) (DsRNA-binding protein 4F.2)
**Q9JIL3**	*ILF3*	*Rattus norvegicus (Rat)*	Chordata	131	29.30%	Interleukin enhancer-binding factor 3
**Q9JIL3-2**	*ILF3*	*Rattus norvegicus (Rat)*	Chordata	131	29.30%	Isoform 2 of Interleukin enhancer-binding factor 3
**Q9Z1X4-2**	*ILF3*	*Mus musculus (Mouse)*	Chordata	130	30.90%	Isoform 2 of Interleukin enhancer-binding factor 3
**Q9Z1X4**	*ILF3*	*Mus musculus (Mouse)*	Chordata	130	30.90%	Interleukin enhancer-binding factor 3
**Q9Z1X4-3**	*ILF3*	*Mus musculus (Mouse)*	Chordata	130	30.90%	Isoform 3 of Interleukin enhancer-binding factor 3
**B9LB70**	*RNC*	*Chloroflexus aurantiacus (strain ATCC 29364 / DSM 637 / Y-400-fl)*	Chloroflexi	128	42.40%	Ribonuclease 3 (EC 3.1.26.3) (Ribonuclease III) (RNase III)
**A9WJ69**	*RNC*	*Chloroflexus aurantiacus*	Chloroflexi	128	42.40%	Ribonuclease 3 (EC 3.1.26.3) (Ribonuclease III) (RNase III)
**A5V230**	*RNC*	*Roseiflexus sp. (strain RS-1)*	Chloroflexi	127	36.60%	Ribonuclease 3 (EC 3.1.26.3) (Ribonuclease III) (RNase III)
**Q6NXA4**	*ILF3*	*Danio rerio (Zebrafish) (Brachydanio rerio)*	Chordata	125	25.60%	Interleukin enhancer-binding factor 3 homolog
**Q9JKU6**	*STRBP*	*Rattus norvegicus (Rat)*	Chordata	124	28.20%	Spermatid perinuclear RNA-binding protein (74 kDa double-stranded RNA-binding protein) (p74)
**B0USS1**	*RNC*	*Histophilus somni (Haemophilus somnus)*	Proteobacteria	124	32.90%	Ribonuclease 3 (EC 3.1.26.3) (Ribonuclease III) (RNase III)
**B0BUA6**	*RNC*	*Actinobacillus pleuropneumoniae (Haemophilus pleuropneumoniae)*	Proteobacteria	124	34.50%	Ribonuclease 3 (EC 3.1.26.3) (Ribonuclease III) (RNase III)
**A3MZQ9**	*RNC*	*Actinobacillus pleuropneumoniae*	Proteobacteria	124	34.50%	Ribonuclease 3 (EC 3.1.26.3) (Ribonuclease III) (RNase III)

		(Haemophilus pleuropneumoniae)				
**A7NGC5**	*RNC*	*Roseiflexus castenholzii*	Chloroflexi	123	37.40%	Ribonuclease 3 (EC 3.1.26.3) (Ribonuclease III) (RNase III)
**C6DC00**	*RNC*	*Pectobacterium carotovorum (Erwinia carotovora)*	Proteobacteria	122	37.70%	Ribonuclease 3 (EC 3.1.26.3) (Ribonuclease III) (RNase III)
**Q6D219**	*RNC*	*Pectobacterium atrosepticum (Erwinia carotovora subsp. atroseptica)*	Proteobacteria	122	37.70%	Ribonuclease 3 (EC 3.1.26.3) (Ribonuclease III) (RNase III)
**Q8CJ67-4**	*STAU2*	*Mus musculus (Mouse)*	Chordata	121	35.80%	Isoform 4 of Double-stranded RNA-binding protein Staufen homolog 2
**B2VI46**	*RNC*	*Erwinia tasmaniensis*	Proteobacteria	121	35.10%	Ribonuclease 3 (EC 3.1.26.3) (Ribonuclease III) (RNase III)
**Q9NII1-5**	*ADAR*	*Drosophila melanogaster (Fruit fly)*	Arthropoda	120	27.90%	Isoform F of Double-stranded RNA-specific editase Adar
**Q9NII1-7**	*ADAR*	*Drosophila melanogaster (Fruit fly)*	Arthropoda	120	27.90%	Isoform E of Double-stranded RNA-specific editase Adar
**Q9NII1-4**	*ADAR*	*Drosophila melanogaster (Fruit fly)*	Arthropoda	120	27.90%	Isoform D of Double-stranded RNA-specific editase Adar
**Q9NII1**	*ADAR*	*Drosophila melanogaster (Fruit fly)*	Arthropoda	120	27.90%	Double-stranded RNA-specific editase Adar (EC 3.5.-.-) (Adenosine deaminase that act on RNA) (Pre-mRNA adenosine deaminase) (RNA-editing deaminase 1) (RNA-editing enzyme 1) (dADAR) (dsRNA adenosine deaminase)
**B8GAM6**	*RNC*	*Chloroflexus aggregans*	Chloroflexi	120	39.10%	Ribonuclease 3 (EC 3.1.26.3) (Ribonuclease III) (RNase III)
**A8GI25**	*RNC*	*Serratia proteamaculans*	Proteobacteria	120	35.10%	Ribonuclease 3 (EC 3.1.26.3) (Ribonuclease III) (RNase III)
**B8D7F5**	*RNC*	*Buchnera aphidicola*	Proteobacteria	120	33.80%	Ribonuclease 3 (EC 3.1.26.3) (Ribonuclease III) (RNase III)
**P57346**	*RNC*	*Buchnera aphidicola*	Proteobacteria	120	33.80%	Ribonuclease 3 (EC 3.1.26.3) (Ribonuclease III) (RNase III)
**B8D951**	*RNC*	*Buchnera aphidicola*	Proteobacteria	120	33.80%	Ribonuclease 3 (EC 3.1.26.3) (Ribonuclease III) (RNase III)
**Q8K9R1**	*RNC*	*Buchnera aphidicola*	Proteobacteria	119	35.10%	Ribonuclease 3 (EC 3.1.26.3) (Ribonuclease III) (RNase III)
**Q492D1**	*RNC*	*Candidatus Blochmannia pennsylvanicus*	Proteobacteria	119	36.00%	Ribonuclease 3 (EC 3.1.26.3) (Ribonuclease III) (RNase III)
**Q9NS39**	*RED2*	*Homo sapiens (Human)*	Chordata	117	26.90%	Double-stranded RNA-specific editase B2 (EC 3.5.-.-) (RNA-dependent adenosine deaminase 3) (RNA-editing deaminase 2) (RNA-editing enzyme 2) (dsRNA adenosine deaminase B2)
**Q57LD0**	*RNC*	*Salmonella choleraesuis*	Proteobacteria	117	35.10%	Ribonuclease 3 (EC 3.1.26.3) (Ribonuclease III) (RNase III)
**D4GKM1**	*RNC*	*Pantoea ananas (Erwinia uredovora)*	Proteobacteria	117	35.10%	Ribonuclease 3 (EC 3.1.26.3) (Ribonuclease III) (RNase III)
**Q608M7**	*RNC*	*Methylococcus capsulatus*	Proteobacteria	117	39.10%	Ribonuclease 3 (EC 3.1.26.3) (Ribonuclease III) (RNase III)

Table S4. Detailed sequence information of PACT members used for constructing a phylogenetic tree.

Entry	Gene names	Taxonomic lineage (SPECIES)	Taxonomic lineage (PHYLUM)	Score	Identity	Protein names
**O75569**	*PRKRA*	*Homo sapiens (Human)*	Chordata	1,637	100.00%	Interferon-inducible double-stranded RNA-dependent protein kinase activator A (PKR-associated protein X) (PKR-associating protein X) (Protein activator of the interferon-induced protein kinase) (Protein kinase, interferon-inducible double-stranded RNA-dependent activator)
**Q9WTX2**	*PRKRA*	*Mus musculus (Mouse)*	Chordata	1,617	98.10%	Interferon-inducible double-stranded RNA-dependent protein kinase activator A (PKR-associated protein X) (PKR-associating protein X) (RAX) (Protein activator of the interferon-induced protein kinase) (Protein kinase, interferon-inducible double-stranded RNA-dependent activator)
**Q4V8C7**	*PRKRA*	*Rattus norvegicus (Rat)*	Chordata	1,611	97.80%	Interferon-inducible double-stranded RNA-dependent protein kinase activator A (Protein activator of the interferon-induced protein kinase) (Protein kinase, interferon-inducible double-stranded RNA-dependent activator)
**Q2HJ92**	*PRKRA*	*Bos taurus (Bovine)*	Chordata	1,611	98.10%	Interferon-inducible double-stranded RNA-dependent protein kinase activator A (Protein activator of the interferon-induced protein kinase) (Protein kinase, interferon-inducible double-stranded RNA-dependent activator)
**O75569-2**	*PRKRA*	*Homo sapiens (Human)*	Chordata	1,530	99.30%	Isoform 2 of Interferon-inducible double-stranded RNA-dependent protein kinase activator A
**O75569-3**	*PRKRA*	*Homo sapiens (Human)*	Chordata	1,509	100.00%	Isoform 3 of Interferon-inducible double-stranded RNA-dependent protein kinase activator A
**Q7ZYA5**	*PRKRAA*	*Xenopus laevis (African clawed frog)*	Chordata	964	61.10%	Interferon-inducible double-stranded RNA-dependent protein kinase activator A homolog A
**Q91836**	*PRKRAB*	*Xenopus laevis (African clawed frog)*	Chordata	953	62.50%	Interferon-inducible double-stranded RNA-dependent protein kinase activator A homolog B (Double-stranded RNA-binding protein A) (XlRBPA)
**Q5BJ52**	*TARBP2*	*Xenopus tropicalis (Western clawed frog) (Silurana tropicalis)*	Chordata	730	45.10%	RISC-loading complex subunit tarbp2
**B0V3F8**	*PRKRA*	*Danio rerio (Zebrafish) (Brachydanio rerio)*	Chordata	729	50.70%	Interferon-inducible double-stranded RNA-dependent protein kinase activator A homolog
**Q6GPZ1**	*TARBP2*	*Xenopus laevis (African clawed frog)*	Chordata	714	44.50%	RISC-loading complex subunit tarbp2
**Q7SXR1**	*TARBP2*	*Danio rerio (Zebrafish) (Brachydanio rerio)*	Chordata	705	44.90%	RISC-loading complex subunit tarbp2
**Q4SS66**	*TARBP2*	*Tetraodon nigroviridis (Spotted green pufferfish) (Chelonodon nigroviridis)*	Chordata	704	45.40%	RISC-loading complex subunit tarbp2
**Q0IIG6**	*TARBP2*	*Bos taurus (Bovine)*	Chordata	666	43.30%	RISC-loading complex subunit TARBP2
**Q15633**	*TARBP2*	*Homo sapiens (Human)*	Chordata	665	41.90%	RISC-loading complex subunit TARBP2 (TAR RNA-binding protein 2) (Trans-activation-responsive RNA-binding protein)

**P97473**	*TARBP2*	*Mus musculus (Mouse)*	Chordata	662	42.20%	RISC-loading complex subunit TARBP2 (Protamine-1 RNA-binding protein) (PRM-1 RNA-binding protein) (TAR RNA-binding protein 2)
**Q15633-2**	*TARBP2*	*Homo sapiens (Human)*	Chordata	658	42.60%	Isoform 2 of RISC-loading complex subunit TARBP2 (Isoform TRBP1 of RISC-loading complex subunit TARBP2)
**Q3SWU0**	*TARBP2*	*Rattus norvegicus (Rat)*	Chordata	656	41.90%	RISC-loading complex subunit TARBP2
**Q5M7M7**	*STAU2*	*Xenopus tropicalis (Western clawed frog) (Silurana tropicalis)*	Chordata	203	32.00%	Double-stranded RNA-binding protein Staufen homolog 2
**Q7ZW47**	*STAU2*	*Danio rerio (Zebrafish) (Brachydanio rerio)*	Chordata	195	34.30%	Double-stranded RNA-binding protein Staufen homolog 2
**Q8CJ67-3**	*STAU2*	*Mus musculus (Mouse)*	Chordata	194	26.90%	Isoform 3 of Double-stranded RNA-binding protein Staufen homolog 2
**Q8CJ67-2**	*STAU2*	*Mus musculus (Mouse)*	Chordata	194	26.90%	Isoform 2 of Double-stranded RNA-binding protein Staufen homolog 2 (Isoform Short of Double-stranded RNA-binding protein Staufen homolog 2)
**Q8CJ67**	*STAU2*	*Mus musculus (Mouse)*	Chordata	194	26.90%	Double-stranded RNA-binding protein Staufen homolog 2
**Q9NUL3-4**	*STAU2*	*Homo sapiens (Human)*	Chordata	192	26.50%	Isoform 4 of Double-stranded RNA-binding protein Staufen homolog 2
**Q9NUL3-6**	*STAU2*	*Homo sapiens (Human)*	Chordata	192	26.50%	Isoform 6 of Double-stranded RNA-binding protein Staufen homolog 2
**Q9NUL3-8**	*STAU2*	*Homo sapiens (Human)*	Chordata	192	26.50%	Isoform 8 of Double-stranded RNA-binding protein Staufen homolog 2
**Q9NUL3-3**	*STAU2*	*Homo sapiens (Human)*	Chordata	192	26.50%	Isoform 3 of Double-stranded RNA-binding protein Staufen homolog 2
**Q9NUL3-7**	*STAU2*	*Homo sapiens (Human)*	Chordata	192	26.50%	Isoform 7 of Double-stranded RNA-binding protein Staufen homolog 2
**Q9NUL3-2**	*STAU2*	*Homo sapiens (Human)*	Chordata	192	26.50%	Isoform 2 of Double-stranded RNA-binding protein Staufen homolog 2 (Isoform Short of Double-stranded RNA-binding protein Staufen homolog 2)
**Q9NUL3**	*STAU2*	*Homo sapiens (Human)*	Chordata	192	26.50%	Double-stranded RNA-binding protein Staufen homolog 2
**Q68SB1-3**	*STAU2*	*Rattus norvegicus (Rat)*	Chordata	189	32.60%	Isoform 3 of Double-stranded RNA-binding protein Staufen homolog 2 (Isoform SS of Double-stranded RNA-binding protein Staufen homolog 2)
**Q68SB1-4**	*STAU2*	*Rattus norvegicus (Rat)*	Chordata	189	32.60%	Isoform 4 of Double-stranded RNA-binding protein Staufen homolog 2 (Isoform LS of Double-stranded RNA-binding protein Staufen homolog 2) (Isoform B of Double-stranded RNA-binding protein Staufen homolog 2)
**Q68SB1-2**	*STAU2*	*Rattus norvegicus (Rat)*	Chordata	189	32.60%	Isoform 2 of Double-stranded RNA-binding protein Staufen homolog 2 (Isoform SL of Double-stranded RNA-binding protein Staufen homolog 2)
**Q68SB1**	*STAU2*	*Rattus norvegicus (Rat)*	Chordata	189	32.60%	Double-stranded RNA-binding protein Staufen homolog 2 (r-staufen protein)
**O95793-2**	*STAU1*	*Homo sapiens (Human)*	Chordata	180	32.10%	Isoform Short of Double-stranded RNA-binding protein Staufen homolog 1
**O95793**	*STAU1*	*Homo sapiens (Human)*	Chordata	180	32.10%	Double-stranded RNA-binding protein Staufen homolog 1
**D2GVP7**	*STAU1*	*Ailuropoda melanoleuca (Giant panda)*	Chordata	171	31.80%	Double-stranded RNA-binding protein Staufen homolog 1
**Q9Z108**	*STAU1*	*Mus musculus (Mouse)*	Chordata	169	31.80%	Double-stranded RNA-binding protein Staufen homolog 1
**O95793-3**	*STAU1*	*Homo sapiens (Human)*	Chordata	167	30.70%	Isoform 3 of Double-stranded RNA-binding protein Staufen homolog 1

**Q3L1C9**	*STAUH*	*Aplysia californica (California sea hare)*	Mollusca	166	28.60%	Double-stranded RNA-binding protein Staufen homolog
**P25159-2**	*STAU*	*Drosophila melanogaster (Fruit fly)*	Arthropoda	165	31.70%	Isoform B of Maternal effect protein staufen
**P25159**	*STAU*	*Drosophila melanogaster (Fruit fly)*	Arthropoda	165	31.70%	Maternal effect protein staufen
**B9LB70**	*RNC*	*Chloroflexus aurantiacus (strain ATCC 29364 / DSM 637 / Y-400-fl)*	Chloroflexi	135	45.50%	Ribonuclease 3 (EC 3.1.26.3) (Ribonuclease III) (RNase III)
**A9WJ69**	*RNC*	*Chloroflexus aurantiacus*	Chloroflexi	135	45.50%	Ribonuclease 3 (EC 3.1.26.3) (Ribonuclease III) (RNase III)
**P55265-3**	*DSRAD*	*Homo sapiens (Human)*	Chordata	132	29.40%	Isoform 3 of Double-stranded RNA-specific adenosine deaminase (Isoform ADAR-c of Double-stranded RNA-specific adenosine deaminase)
**B8GAM6**	*RNC*	*Chloroflexus aggregans*	Chloroflexi	124	43.90%	Ribonuclease 3 (EC 3.1.26.3) (Ribonuclease III) (RNase III)
**Q99MU3-4**	*DSRAD*	*Mus musculus (Mouse)*	Chordata	124	29.00%	Isoform 4 of Double-stranded RNA-specific adenosine deaminase (Isoform ADAR1Sb of Double-stranded RNA-specific adenosine deaminase) (Isoform p80 of Double-stranded RNA-specific adenosine deaminase)
**Q99MU3-5**	*DSRAD*	*Mus musculus (Mouse)*	Chordata	124	29.00%	Isoform 5 of Double-stranded RNA-specific adenosine deaminase (Isoform p110 of Double-stranded RNA-specific adenosine deaminase)
**P55266**	*DSRAD*	*Rattus norvegicus (Rat)*	Chordata	124	29.00%	Double-stranded RNA-specific adenosine deaminase (DRADA) (EC 3.5.4.37)
**Q99MU3**	*DSRAD*	*Mus musculus (Mouse)*	Chordata	124	29.00%	Double-stranded RNA-specific adenosine deaminase (DRADA) (EC 3.5.4.37) (RNA adenosine deaminase 1)
**Q99MU3-3**	*DSRAD*	*Mus musculus (Mouse)*	Chordata	117	29.40%	Isoform 3 of Double-stranded RNA-specific adenosine deaminase (Isoform ADAR1Sa of Double-stranded RNA-specific adenosine deaminase)
**Q99MU3-2**	*DSRAD*	*Mus musculus (Mouse)*	Chordata	117	29.40%	Isoform 2 of Double-stranded RNA-specific adenosine deaminase (Isoform ADAR1La of Double-stranded RNA-specific adenosine deaminase)
**Q0DKP4**	*DRB2*	*Oryza sativa (Rice)*	Streptophyta	117	34.50%	Double-stranded RNA-binding protein 2 (dsRNA-binding protein 2) (dsRNA-binding protein 3) (OsDRB3)
**C4K3Z3**	*RNC*	*Candidatus Hamiltonella defensa*	Proteobacteria	116	39.40%	Ribonuclease 3 (EC 3.1.26.3) (Ribonuclease III) (RNase III)
**P51833**	*RNC*	*Bacillus subtilis*	Firmicutes	110	43.50%	Ribonuclease 3 (EC 3.1.26.3) (Ribonuclease III) (RNase III)
**Q7VL75**	*RNC*	*Haemophilus ducreyi*	Proteobacteria	110	33.80%	Ribonuclease 3 (EC 3.1.26.3) (Ribonuclease III) (RNase III)
**Q91FI4**	*340R*	*Invertebrate iridescent virus 6 (IIV-6) (Chilo iridescent virus)*	Iridoviridae	107	31.80%	DRBM domain-containing protein 340R

Table S5.Detailed sequence information of GW182 members used for constructing a phylogenetic tree.

Entry	Gene names	Taxonomic lineage (SPECIES)	Taxonomic lineage (PHYLUM)	Score	Identity	Protein names
**Q8SY33**	*GW182*	*Drosophila melanogaster (Fruit fly)*	Arthropoda	7,295	100.00%	Protein Gawky
**Q8SY33-3**	*GW182*	*Drosophila melanogaster (Fruit fly)*	Arthropoda	7,270	99.90%	Isoform J of Protein Gawky
**Q8SY33-2**	*GW182*	*Drosophila melanogaster (Fruit fly)*	Arthropoda	7,266	99.80%	Isoform I of Protein Gawky
**Q3UHC0**	*TNRC6C*	*Mus musculus (Mouse)*	Chordata	731	26.80%	Trinucleotide repeat-containing gene 6C protein
**Q9HCJ0**	*TNRC6C*	*Homo sapiens (Human)*	Chordata	730	26.50%	Trinucleotide repeat-containing gene 6C protein
**Q9HCJ0-2**	*TNRC6C*	*Homo sapiens (Human)*	Chordata	729	26.50%	Isoform 2 of Trinucleotide repeat-containing gene 6C protein
**Q9UPQ9**	*TNRC6B*	*Homo sapiens (Human)*	Chordata	652	27.10%	Trinucleotide repeat-containing gene 6B protein
**Q3UHK8**	*TNRC6A*	*Mus musculus (Mouse)*	Chordata	649	27.40%	Trinucleotide repeat-containing gene 6A protein
**Q8NDV7-2**	*TNRC6A*	*Homo sapiens (Human)*	Chordata	645	26.70%	Isoform 2 of Trinucleotide repeat-containing gene 6A protein
**Q8NDV7-5**	*TNRC6A*	*Homo sapiens (Human)*	Chordata	645	26.70%	Isoform 5 of Trinucleotide repeat-containing gene 6A protein
**Q8NDV7**	*TNRC6A*	*Homo sapiens (Human)*	Chordata	645	26.70%	Trinucleotide repeat-containing gene 6A protein (CAG repeat protein 26) (EMSY interactor protein) (GW182 autoantigen) (Protein GW1) (Glycine-tryptophan protein of 182 kDa)
**Q8NDV7-6**	*TNRC6A*	*Homo sapiens (Human)*	Chordata	623	26.60%	Isoform 6 of Trinucleotide repeat-containing gene 6A protein
**Q8BKI2**	*TNRC6B*	*Mus musculus (Mouse)*	Chordata	612	26.90%	Trinucleotide repeat-containing gene 6B protein
**Q9UPQ9-1**	*TNRC6B*	*Homo sapiens (Human)*	Chordata	549	25.50%	Isoform 2 of Trinucleotide repeat-containing gene 6B protein
**Q9UPQ9-2**	*TNRC6B*	*Homo sapiens (Human)*	Chordata	503	28.60%	Isoform 3 of Trinucleotide repeat-containing gene 6B protein

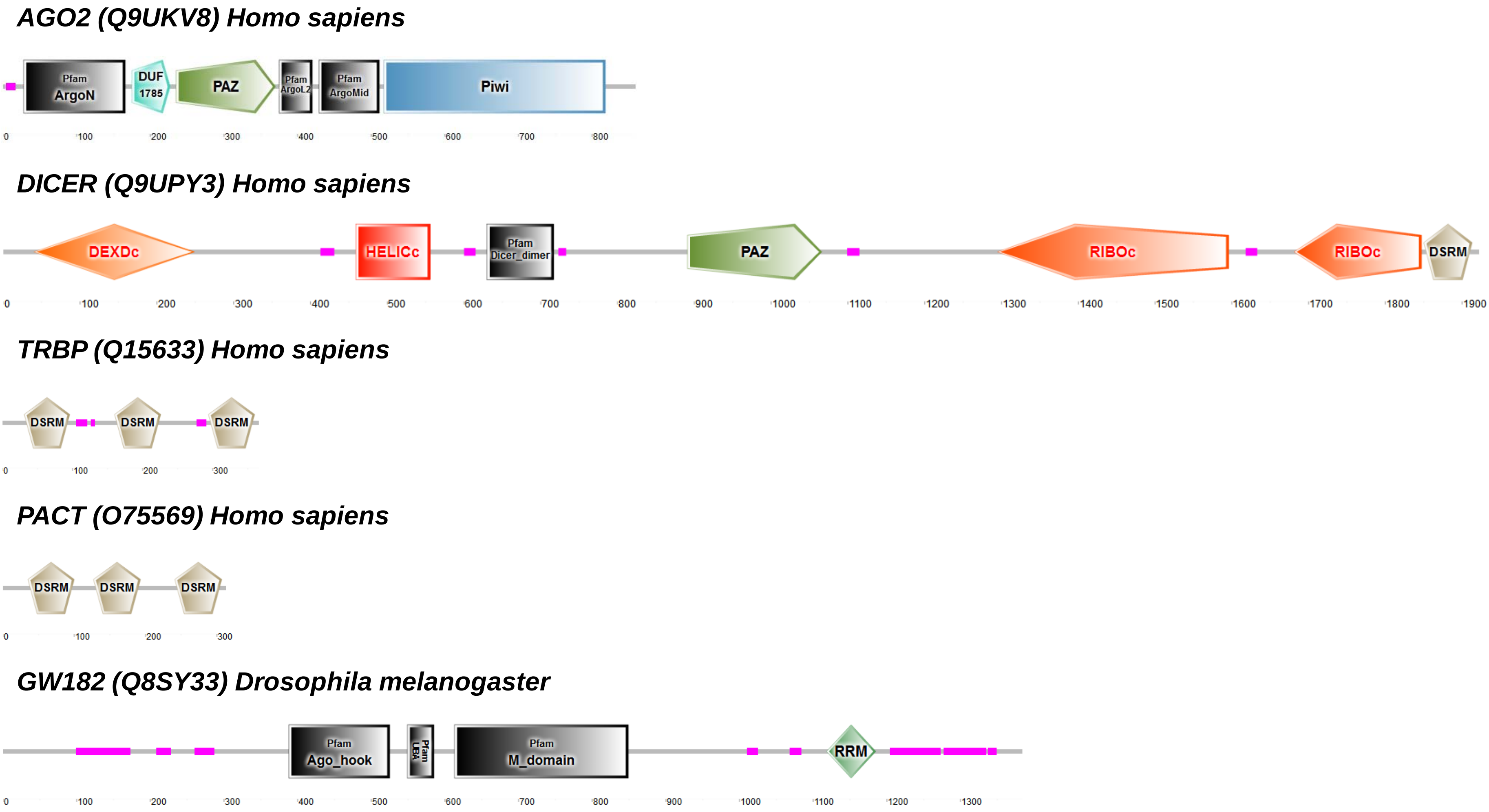

Figure S1. Conserved domains of the major RISC members (AGO2, DICER, TRBP, PACT and GW182).

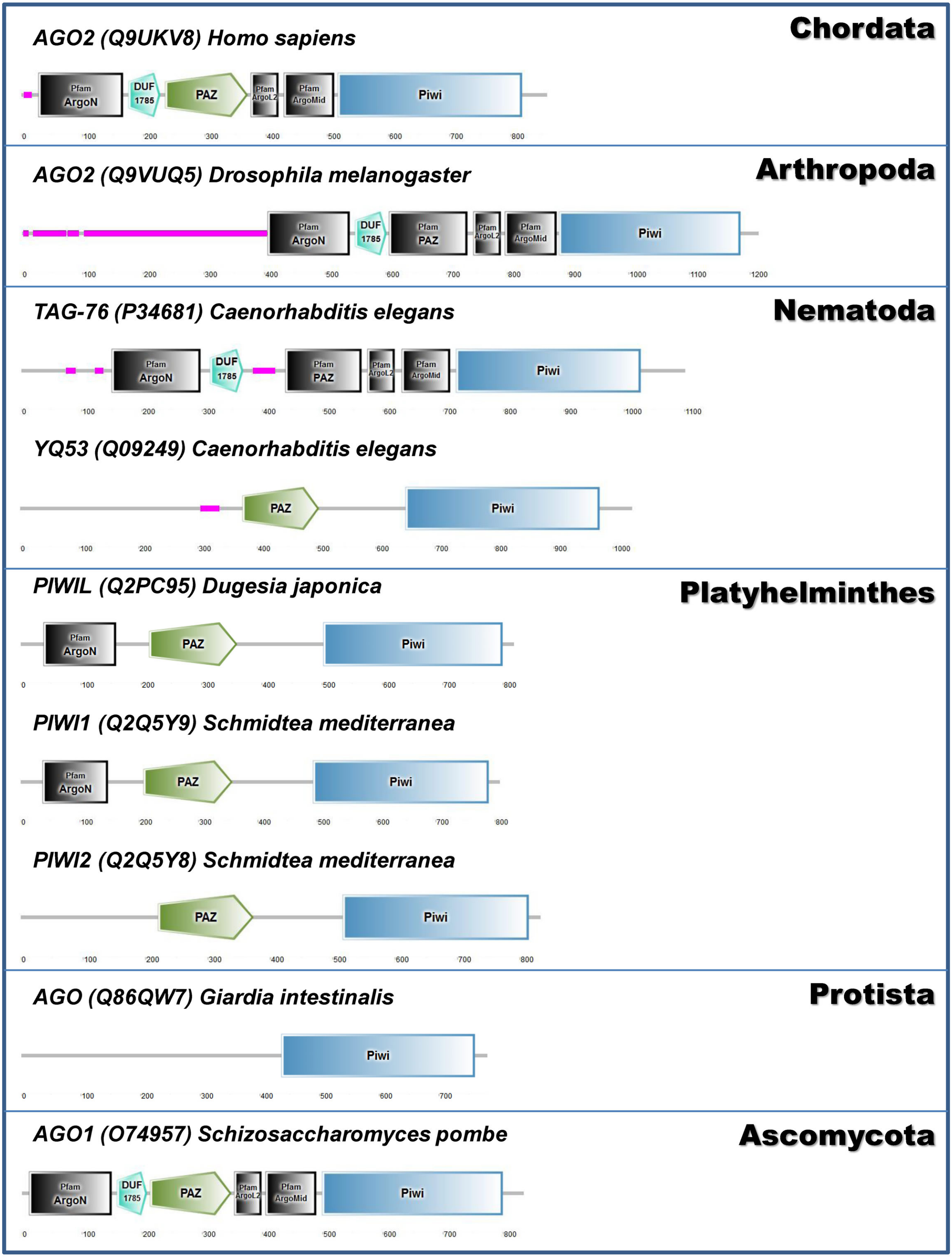

Figure S2. Conserved domains of AGO members in the different species.

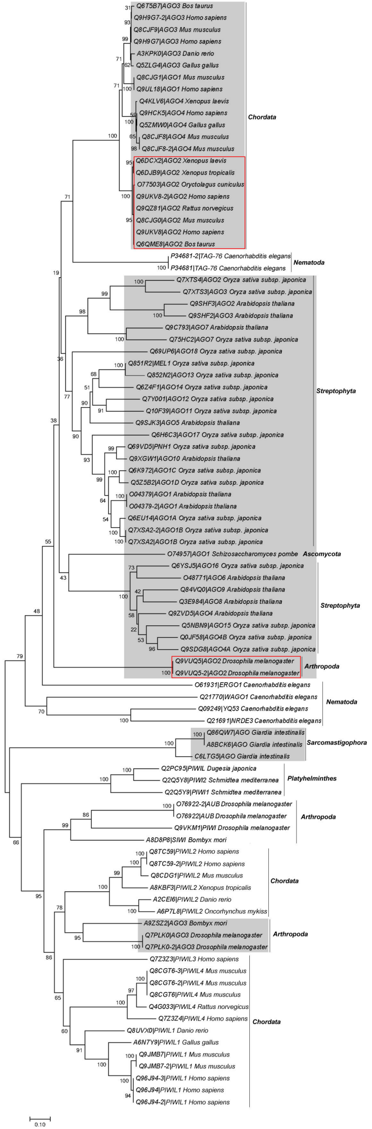

Figure S3. Phylogenetic tree of AGO members.

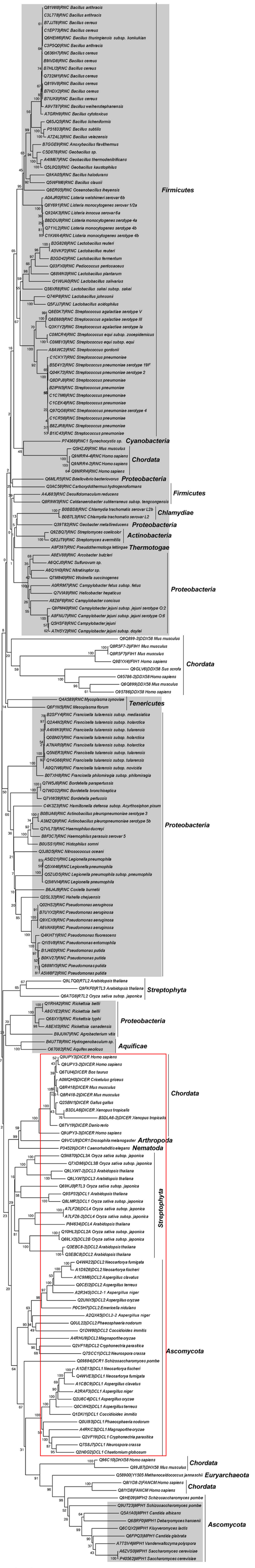

Figure S4. Phylogenetic tree of DICER members.

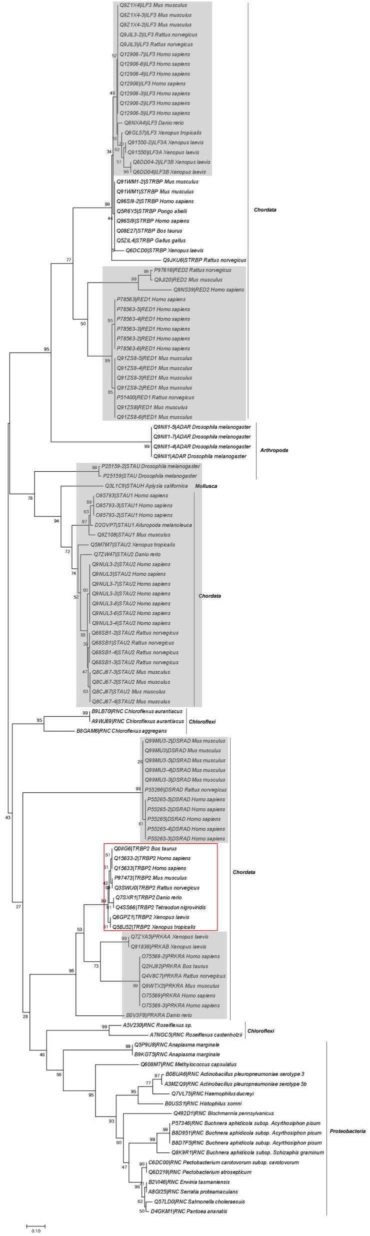

Figure S5. Phylogenetic tree of TRBP members.

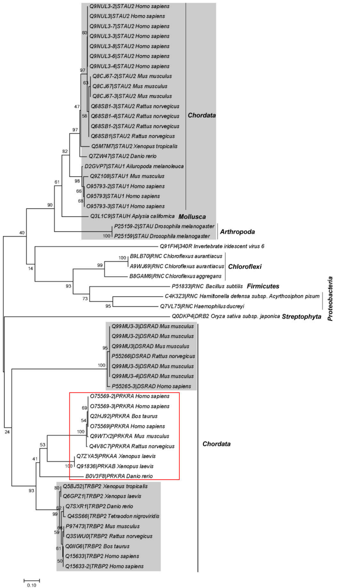

Figure S6. Phylogenetic tree of PACT members.

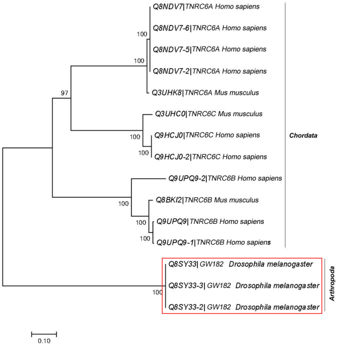

Figure S7. Phylogenetic tree of GW182 members.

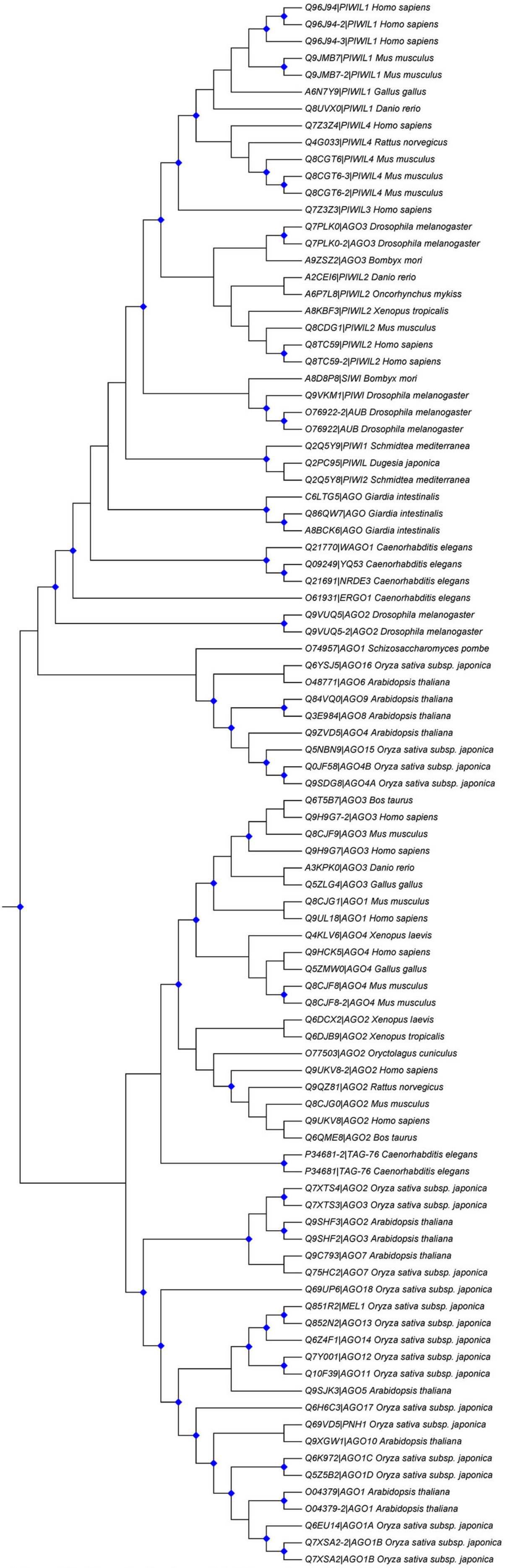

Figure S8. Gene duplications of AGO members. Closed diamonds represent gene duplication events.

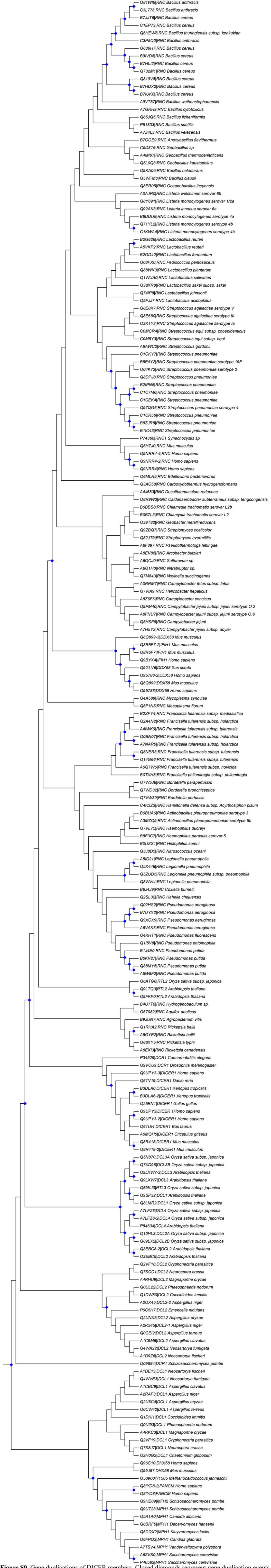

Figure S9. Gene duplications of DICER members. Closed diamonds represent gene duplication events.

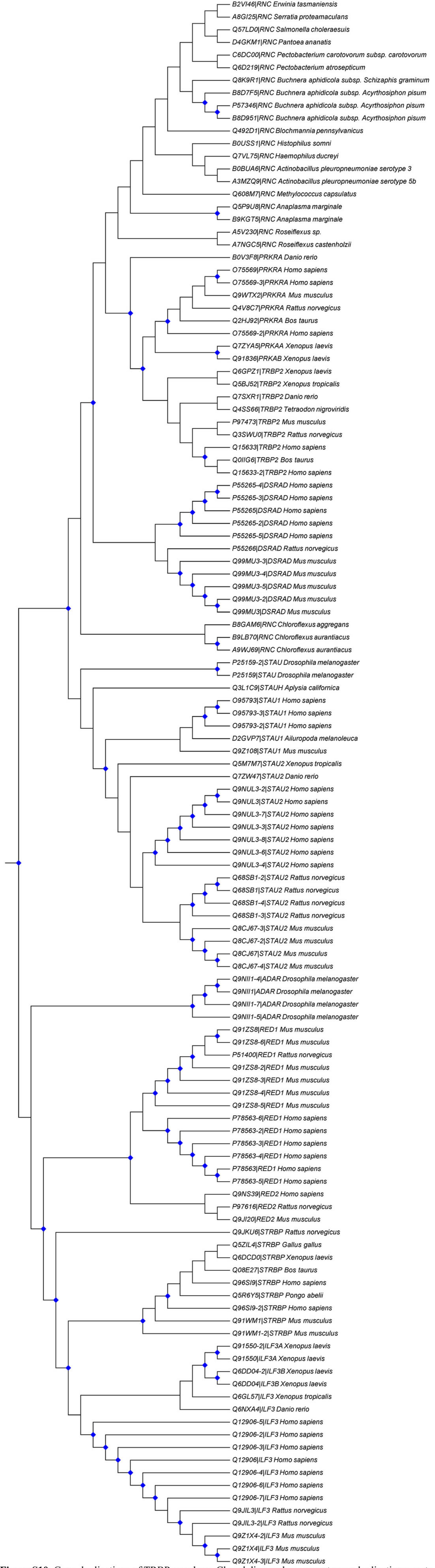

Figure S10. Gene duplications of TRBP members. Closed diamonds represent gene duplication events.

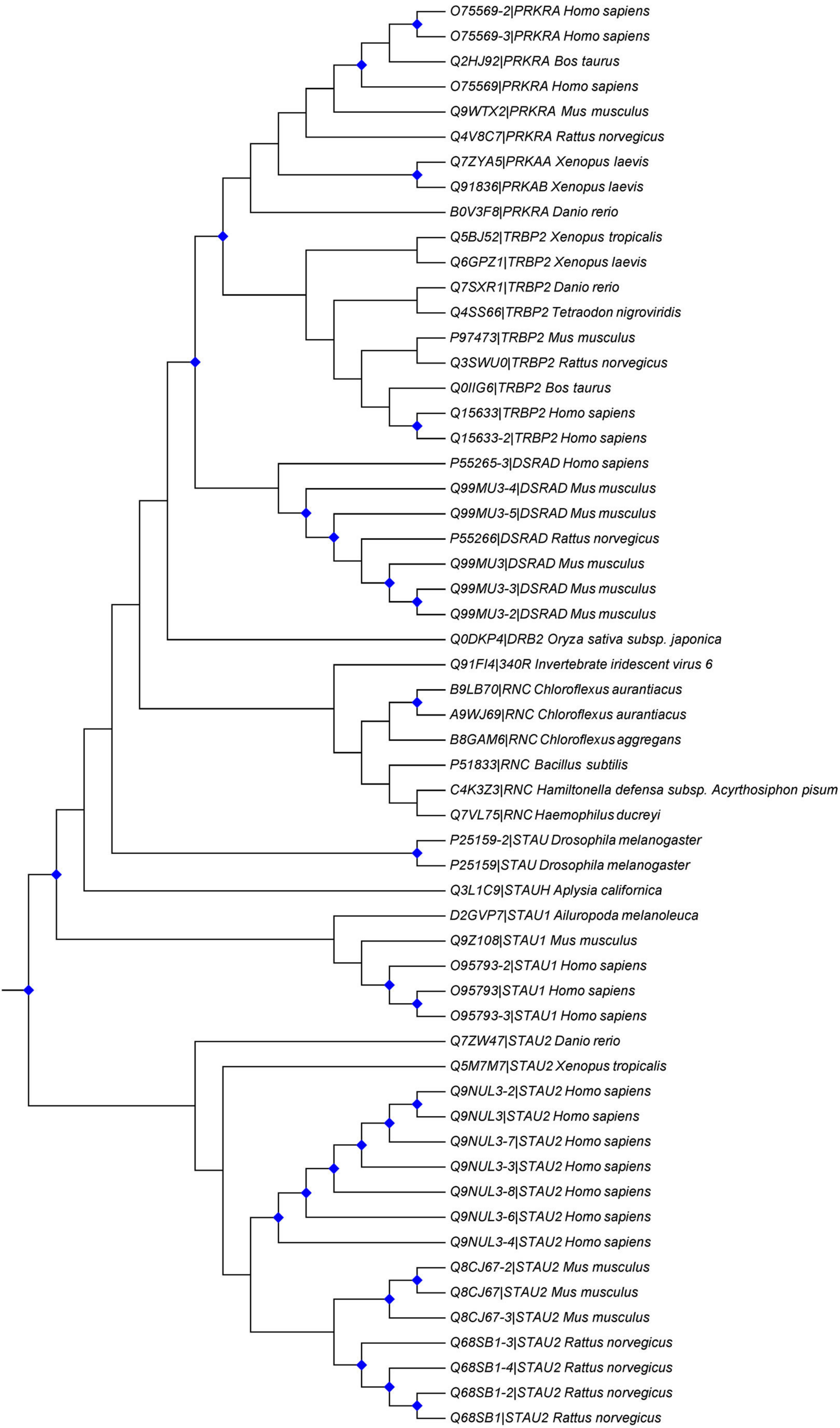

Figure S11. Gene duplications of PACT members. Closed diamonds represent gene duplication events.

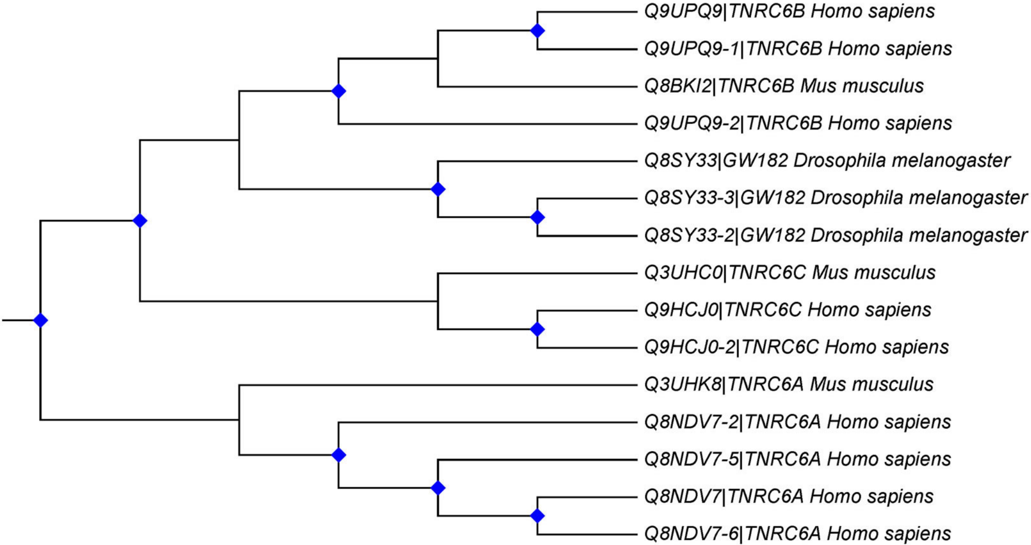

Figure S12. Gene duplications of GW182 members. Closed diamonds represent gene duplication events.
